# Identifying the genetic diversity, genetic structure and a core collection of *Ziziphus jujuba* Mill. var. jujuba accessions using microsatellite markers

**DOI:** 10.1038/srep31503

**Published:** 2016-08-17

**Authors:** Chaoqun Xu, Jiao Gao, Zengfeng Du, Dengke Li, Zhe Wang, Yingyue Li, Xiaoming Pang

**Affiliations:** 1 National Engineering Laboratory for Tree Breeding, Key Laboratory of Genetics and Breeding in Forest Trees and Ornamental Plants, Ministry of Education, College of Biological Sciences and Biotechnology, Beijing Forestry University, Beijing (100083), China; 2 National Foundation for Improved Cultivar of Chinese Jujube, Cangzhou Heibei, 061000, China; 3 Pomology Institute, Shanxi Academy of Agricultural Science, Taigu, Shanxi 030815, China.

## Abstract

*Ziziphus* is a genus of spiny shrubs and small trees in the Rhamnaceae family. This group has a controversial taxonomy, with more than 200 species described, including Chinese jujube (*Ziziphus jujuba* Mill. var. jujuba) and Indian jujube (*Z. mauritiana*), as well as several other important cultivated fruit crops. Using 24 SSR markers distributed across the Chinese jujube genome, 962 jujube accessions from the two largest germplasm repositories were genotyped with the aim of analyzing the genetic diversity and structure and constructing a core collection that retain high genetic diversity. A molecular profile comparison revealed 622 unique genotypes, among which 123 genotypes were genetically identical to at least one other accessions. STRUCTURE analysis and multivariate analyses (Cluster and PCoA) roughly divided the accessions into three major groups, with some admixture among groups. A simulated annealing algorithm and a heuristic algorithm were chosen to construct the core collection. A final core of 150 accessions was selected, comprising 15.6% of the analyzed accessions and retaining more than 99.5% of the total alleles detected. We found no significant differences in allele frequency distributions or in genetic diversity parameters between the chosen core accessions and the 622 genetically unique accessions. This work contributes to the understanding of Chinese jujube diversification and the protection of important germplasm resources.

Chinese jujube (*Ziziphus jujuba* Mill. var. jujuba), which belongs to the buckthorn family (Rhamnaceae), is an important deciduous fruit tree that is typically grown in temperate and subtropical areas. It is indigenous to the middle and lower reaches of the Yellow River of China and was first domesticated 7,700 years ago^1^. Chinese jujube is both consumed as a fruit and used in herbal medicine because it has high vitamin C, cyclic AMP, and mineral content (particularly potassium and iron) as well as biologically active compounds^2^. In addition, it is considered an ideal cash crop for arid and semi-arid areas due to its high tolerance to drought and salinity^1^. Historically, Chinese jujube has a large-scale commercial production in China and South Korea, recently, it has been gradually gaining prominence in Australia, the USA and other countries^3^. Chinese jujube has been cultivated throughout China except the northernmost province, Heilongjiang, and the far southwestern province of Tibet, with a cultivation area of two million ha^1^. The leading provinces in Chinese jujube production are Xinjiang followed by Shannxi, Shanxi, Hebei, Shandong, and Henan, accounting for approximately 90% of the entire yield in China.

Chinese jujube originates from its wildtype, sour jujube (*Z. jujuba* Mill. var. spinosa (Bunge) Hu ex H. F. Chow)^1^, mainly propagated by grafting and suckering, but it can also be propagated using seeds. Crossbreeding of Chinese jujube has proven difficult because of the small flower and low fruit and kernel production in the seed^4^. New cultivars are mainly being developed based on selection from spontaneous somatic mutants (‘sports’) and occasional seedlings. The number of cultivars has increased over time, and more than 900 documented cultivars currently exist in China, with these cultivars having been selected by farmers and breeders. Although most of the Chinese jujube cultivars are diploid (2n = 2× = 24), a few triploids exist. ‘Zanghuangdazao’ was the first triploid cultivar found in nature, and it exhibits considerable genetic variation^5^. ‘Pingguozao’ (recently certified as ‘Jingling No. 1’) was also shown to be a triploid using flow cytometry and chromosome counting^6^.

The two largest Chinese jujube collections are housed at the National Chinese Jujube Germplasm Repository, located in Taigu County, Shanxi Province, and the National Foundation for Improved Cultivar of Chinese Jujube, located in Cang County, Hebei Province. Several other smaller local collections also exist. Due to the ease of asexual propagation and the frequent transport of cultivars between regions, there are a large number of synonyms for some cultivar names. Moreover, mislabeling may also occur in the germplasm collections, which hinders cultivar identification, exploitation, evaluation, and use. Therefore, a noteworthy goal is to characterize current Chinese jujube collections to improve management and utilization.

Traditionally, plant cultivar differentiation was based on morphological characteristics and pedigree information. However, morphological descriptions can possess limitations, as morphology can be influenced by environmental factors and requires skilled assessment^7^. With the advent of molecular marker techniques, DNA fingerprinting has become an important tool used to identify and delineate cultivars and quantify variation within the germplasm. Different types of molecular markers, including random amplified polymorphic DNA (RAPD), amplified fragment length polymorphic DNA (AFLP), sequence-related amplified polymorphism (SRAP), and simple sequence repeats (SSR), have been applied to identify Chinese jujube cultivars, evaluate genetic diversity, and conduct QTL mapping^4^. Among these markers, SSRs have become the genetic marker of choice due to a high reproducibility and ability to identify high levels of genetic polymorphism, co-dominance, broad genome distributions, and genetic diversity. A great amount of SSR markers have been developed for *Z. jujuba*^8^,^9^,^10^ and sour jujube^11^ (*Ziziphus jujuba* var. spinosa), which have been used for linkage map construction (unpublished data) and genetic diversity estimations^4^,^5^. More recently, our research group reported that 76 major cultivars employed in Chinese jujube production exhibited comparatively high genetic diversity based on 31 SSR primer pairs compared with fruit crops like grape and apple^4^. We found that the recorded location distributions of many Chinese jujube cultivars may not represent their actual origin. The genetic diversity of 174 Chinese jujube genotypes was also evaluated using AFLP and SRAP markers^12^. Therefore, considering the large number of germplasm resources, it warrants a more comprehensive understanding of the diversity within the germplasm collections.

Management of large germplasm collections is often costly, time-consuming, and labor-intensive, limiting the breeding capacity and in-depth explorations of the germplasms. Because these collections often contain redundant accessions, so it is urgent to build a core collection, which, as the representative germplasm resources of the entire germplasm collection, preserves the maximum genetic diversity and minimum repetition of a crop species^13^. Therefore, a core collection can improve germplasm selection and evaluation for curators and breeders, while maintaining a core set that representative of the genetic diversity of the entire germplasm collection. This strategy would allow for allelic gene varieties and genotype-phenotype associations to be efficiently mined and assessed.

Here we aimed to (1) develop molecular fingerprints and determine the genetic redundancy in the germplasm accession collections, (2) identify the genetic relationships among these accessions, (3) evaluate the level of genetic diversity in the collections, and (4) construct a suitable core collection to be used as a germplasm resource.

## Results

### Chinese jujube germplasm identity analysis

Twenty-four SSR loci were employed to identify unique genotypes among the 947 diploid accessions. In total, 622 distinct SSR genotypes were detected in the collections (Table S1). In total, 499 accessions had unique multi-locus genotypes (Table S1), and each was represented by only one accession in the collections. The remaining 448 accessions, which accounted for 47.3% of the collections, possessed non-unique SSR profiles and were represented by 123 different genotypes (Table 1). Hereafter, accessions that were genetically identical to at least one other accession are defined as a duplicate set. The 123 duplicate sets were found in 198 and 250 accessions for Cangzhou and Taigu, respectively (Table S2). Within the 123 duplicate sets were three distinct types. The first type represented different strains of the same cultivar in Cangzhou (e.g., ‘Dongzao-103’ and ‘Dongzao-100’). The second type corresponded to an identical name with different ‘-C’ or ‘-T’ suffixes, representing the accessions from Cangzhou or Taigu, respectively (e.g., ‘Mayizao-C’ and ‘Mayizao-T’). The third type was the synonym that contained the largest group listed in group 18, with as many as 37 accessions, including several ‘Xiaozao’ accessions (Table S2). Separate identity analyses for Cangzhou and Taigu are also included in Table S2, which revealed 43 and 64 duplicate sets, respectively.

### Genetic diversity of the subset with unique genotypes

All 24 SSR loci successfully amplified polymorphic and reproducible alleles in 622 genotypes. The 24 SSRs yielded high discriminating capacity, as deduced from the low cumulative identity probability (PI) of 8.6E-19 (Table 2). Private alleles were investigated among the sour jujube, diploid accessions and triploid species. Sour jujube accessions revealed 59 alleles and1 private allele in 24 SSRs, whereas no private alleles were identified for the triploid species.

A total of 215 alleles were detected in the 622 unique diploid genotypes, ranging from 3 alleles at BFU0584 and BFU0733 to 21 alleles at BFU0308, with a mean of 8.96 alleles per locus. The Ne value ranged from 1.35 alleles at locus BFU0521 to 8.97 alleles at locus BFU0308, with an average of 3.15 alleles per locus (Table 3). The allele size varied from 104 bp at locus BFU0574 to 316 bp at locus BFU0377. Of the 215 alleles, 128 were considered rare alleles, occurring at a low frequency (<0.05) in the entire germplasm collections and representing 59.5% of the total alleles. A minimum allele frequency of 0.1% was found for all 24 loci, except BFU0263, BFU0733, BFU0478, BFU1279, BFU0277, BFU0501, and BFU1178. The maximum allele frequency (0.86) was observed for allele 240 at BFU0521. In addition, the mean Ho and He values per locus ranged from 0.25 at BFU0479 to 0.91 at BFU0308 and from 0.26 at BFU0521 to 0.89 at BFU0308. Moreover, the important genetic diversity estimator, polymorphic information content (PIC), revealed high diversity levels for all genotypes, averaging 0.56. Sixteen microsatellite loci were highly polymorphic (PIC > 0.5), ranging from 0.51 at BFU0479 to 0.88 at BFU0308. Eight loci exhibited moderate polymorphic trends (0.25 < PIC < 0.5), ranging from 0.25 at BFU0521 to 0.42 at BFU0478 (Table 3).

### Comparison of genetic diversity between the Cangzhou and Taigu repositories

Of the 947 diploid accessions, 479 were from the Cangzhou repository and 468 were from the Taigu repository. As for the 622 distinct genotypes, Cangzhou and Taigu repositories possessed 362 and 315 genotypes, respectively (Table 1). Thus, Cangzhou had more unique genotypes than did Taigu.

The Cangzhou and Taigu repositories exhibited 196 and 176 alleles, with averages of 8.17 and 7.33 alleles, respectively (Table 1). The number of common alleles was 157. The Cangzhou and Taigu repositories exhibited 39 and 19 private alleles, respectively (Table S3). ‘Cuizaohong’ exhibited four loci (BFU0586, BFU0377, BFU0521 and BFU0564) with five private alleles, followed by ‘Henan-12’ and ‘Cuzao’, which each had three private alleles for the Cangzhou repository. ‘Kashixiaozao’ exhibited the largest number of private alleles, followed by ‘Hanguohongyan’ and ‘Chaoyangmopanzao,’ each of which possessed two loci (BFU0539, BFU0614 and BFU1205, BFU0574, respectively) with private alleles in the Taigu repository. However, the frequencies of the private alleles from both the Cangzhou and Taigu repositories were very low, with average values of 0.003 and 0.002, respectively (Table S3). Accessions with one or more private alleles are listed in Table S4, with Cangzhou and Taigu exhibiting 57 and 23 private alleles, respectively.

The mean expected heterozygosity (He) and observed heterozygosity (Ho) were 0.60 and 0.64 for Taigu, whereas the average values were 0.58 and 0.62 for Cangzhou. The mean PIC in Taigu (0.56) was similar to that of Cangzhou (0.53) (Table 1).

### Population Structure and Principal Coordinate Analysis

**Figure 1:**
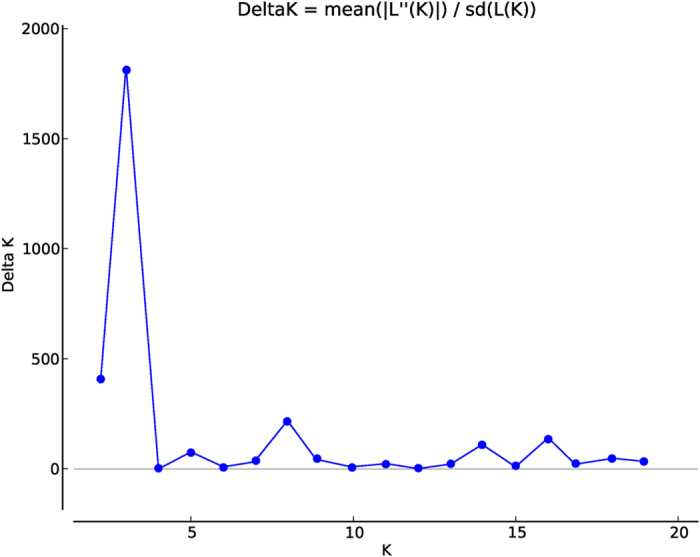
Delta K values for STRUCTURE analysis of Chinese jujube accessions. Delta K, calculated according to Evanno *et al*. 51, is plotted against the number of modeled gene pools (K).

**Figure 2:**
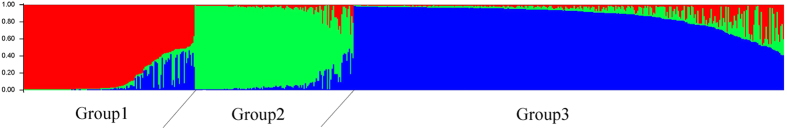
Population structure diagram of the 622 unique genotypes. Note: Vertical lines on the X-axis cannot be clearly marked because of the large number of genotypes. The proportion of each color indicates the probability of each accession being divided into the corresponding group.

In the absence of clear-cut origins of the accessions, a non-stratified strategy was adopted for the genetic structure analysis. Our results showed a clear peak for ΔK at K = 3 (Fig. 1), where all the accessions were roughly divided into three major groups, with some admixture among groups (Fig. 2). About 80% of accessions belonged to each group, which showed strong ancestry values averaging >0.80 (data not shown). Group 3 contained the highest number of accessions (351), followed by group 2 (140) and group 1 (131). Group 1 was comprised almost all of the ‘Dongzao’ accessions, such as ‘Dongzao-40,’ ‘Dongzao-103,’and ‘Chengwudongzao-T.’ Only ‘Gansudongzao’ was included in group 3. All five of the sour jujube accessions were assigned to group 3, albeit in two different nodes. Notably, four accessions from Korea (‘Hanguohongyan,’. ‘Hanguowudeng,’. ‘Hanguojinxiu,’. and ‘Hanguoyuechu’), which were highly adaptable to cold climates, were included in group 2, ‘Hanguofuzao’ from Korea, however, was assigned to group 3, suggesting that it may have a unique ancestry type.

Statistical analysis indicated that the percentage of genotypes with a membership coefficient ≥90% was 63.83%. A total of 83.28% of genotypes exhibited a membership coefficient ≥80%, and only 3.38% of the accessions exhibited a membership coefficient of 5% or less. Based on standard permutation tests of the full data set, the groups defined by *Structure* suggest moderate genetic differentiation, as indicated by the global Fst value of 0.11 (P < 0.01).

**Figure 3:**
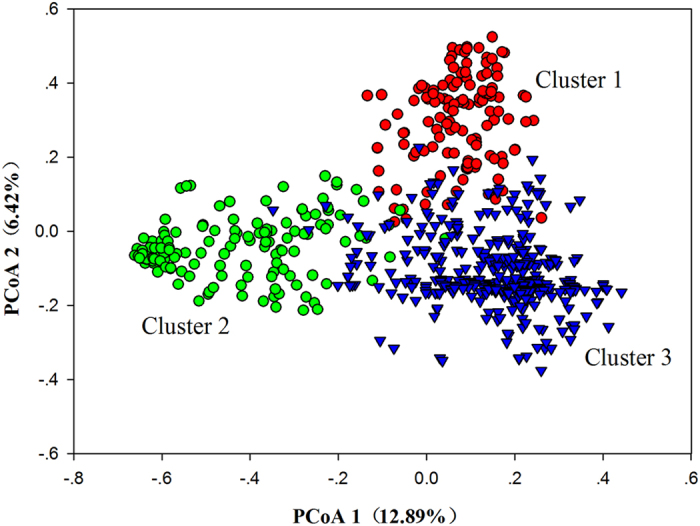
Principal coordinate analysis (PCoA) of the 622 unique genotypes using GenAlEx 6.5 software for 24 SSRs. Note: Accession assignments are depicted with darkred circles, green circles, and blue triangles, respectively.

A principal coordinate analysis (PCoA) roughly divided the 622 unique accessions into three clusters (Fig. 3). Principal coordinates (PCo) 1 and 2 explained 12.9% and 6.4% of the variance in the genotype data, respectively (Fig. 3). More than 50% of the accessions were assigned to cluster 3, whose accessions were much more scattered than those in clusters 1 and 2.

**Figure 4:**
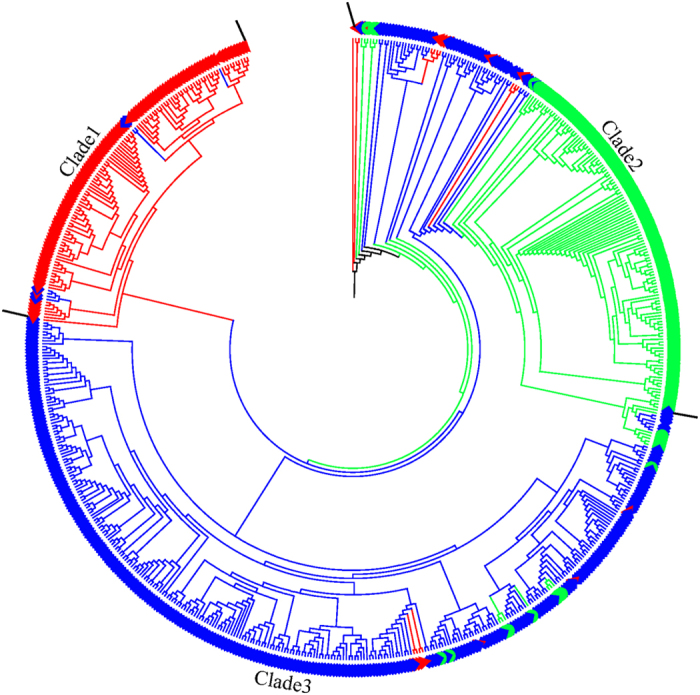
UPGMA dendrogram of the 622 unique genotypes. Note: UPGMA dendrogram was constructed by Powermarker based on frequency-based distance. The three clusters correspond to those of the STRUCTURE groups with the same color.

The dendrogram divided the 622 diploid accessions into three major clades (Fig. 4). Overall, the dendrogram corroborated the *Structure* results, with the exception ofclade 2, in which a few accessions were assigned to group 3.

### Selection of core collections

**Figure 5:**
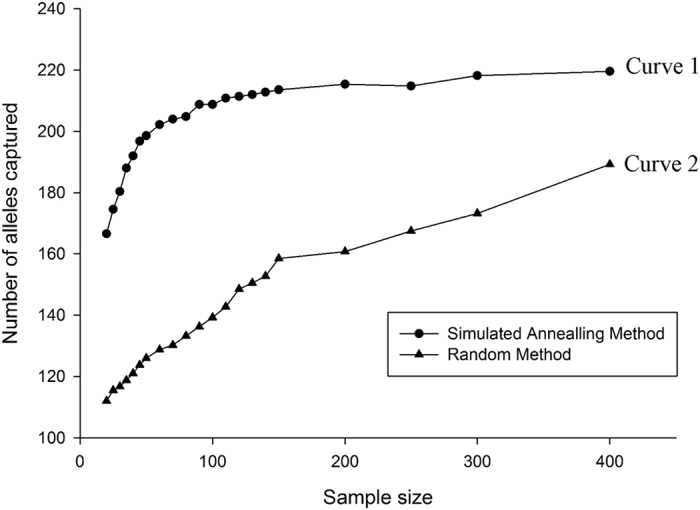
Allele numbers captured as a function of the accession size and included in the core collection based on the simulated annealing algorithm and random algorithm generated using PowerMarker V2.35. Note: Black dots represent the simulated annealing algorithm, and black triangles represent the random algorithm.

To determine the optimal core size, 21 sampling percentages from the whole collection were designed, combined with two sampling strategies. As illustrated in Fig. 5, Curve 2 exhibited inferior efficiency compared to Curve 1, especially for core sets with smaller sample collections, which demonstrated a larger allele retention gap. For instance, the value represented in Curve 1 was approximately 50% higher than the cases in Curve 2 when the core selection size was 20–150, and when the core set reached 400, Curve 2 only captured 85.8% of the total alleles. In contrast, Curve 1 had plateaued as the core set reached 150, and the allelic retention nearly equaled the total alleles in the 947 diploid accessions. Ultimately, the simulated annealing algorithm (represented by Curve 1) is considered the preferred strategy for constructing the core selection, and the core size of 150, which accounted for 15.6% of the total accessions and captured 99.5% of the total alleles, was ultimately defined.

In the present study, we constructed an integrated applied core collection for Chinese jujube that includes 20 retained accessions with applications to genetic research and breeding programs, all of which were chosen based on fruit cracking, fruit size, fruit shape, and commercial importance in the jujube industry (Table S5). Then, a total of 77 and 79 accessions were identified using PowerMarker and PowerCore software at the genotypic level, respectively. All of the 156 accessions were subjected to the relationship test based on the cluster analysis. Finally, a core set of 150 was constructed after deleting 6 duplicates.

**Figure 6:**
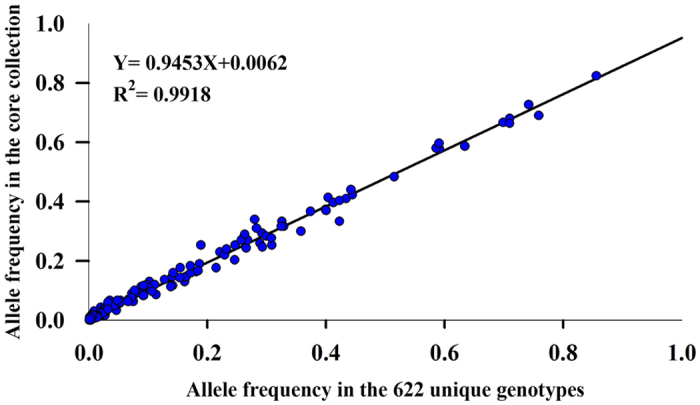
Frequency distribution of the 214 alleles recovered in the core collection (150 genotypes) versus the 622 unique genotypes after analyzing 24 SSR loci. Note: The dots represent the 214 alleles shared by the 150 core collection and the 622 unique genotypes.

The mean values of Na, Ne, Ho, He, and PIC from the core collection were greater than or equal to the 622 diploid accessions (Table 4). Heterozygosity and alleles of all loci in the 150 core collections were 0.64 and 214, respectively, while the 622 diploid accessions yielded values of 0.61 and 215. No significant differences were observed for Na, Ne, Ho, He, and PIC between the core and the 622 diploid accessions, as indicated by Levene’s test for equality of variance and t-tests for equality of means (Table 4). The frequency of alleles in the core collection and the 622 unique genotypes was highly correlated (R = 0.9453) (Fig. 6).

## Discussion

The aim of this work was to identify the genetic diversity, genetic structure, and a core collection of *Ziziphus jujuba* Mill. var jujuba accessions. Now, we interpret our results with regard to genetic diversity and the causes of the genetic redundancy. The present status of genetic structure is briefly discussed. In addition, we further explain the efficiency of the strategy used to construct the core collection. Genetic redundancy is an important issue in plant genetic resource management. The identification of duplicates is important in germplasm repositories, particularly when considering the construction of core collections.

Different rates of duplication have been extensively reported in soybean^14^, lychee^15^, grape^16^ and melon^17^. In the present study, a genetic characterization of Chinese jujube found 123 genotypes that were genetically identical to at least one other accession, which accounts for about half of the collection (47.3%, 947 accessions) (Table S2). This suggests that duplicates may frequently occur in Chinese jujube. Some duplicates (79 groups, e.g., Changmuzao-C’/‘Changmuzao-T’; Table S2) correspond to an accession with an identical name with different suffixes, ‘-C’ and ‘-T’, which represent the accessions from Cangzhou and Taigu, respectively. This may indicate common accessions in both repositories. Other duplicates appear in either of the two germplasm repositories (For example, ‘Yuanlizao-C1’/‘Yuanlizao-C2,’ ‘Jinsi No. 3-T1’/‘Jinsi No. 3-T2,’ etc., Table S2). Some other duplicates may have occurred as a result of incorrect origin identification, as the recorded location of many Chinese jujube cultivars may not represent their actual origin^4^. Olive and grape crops, which have long cultivation histories, have faced similar origin identification issues^18^,^19^. In general, the redundant genotypes are consistent with expectations. This is probably because the sports (spontaneous somatic mutants) or clonal selections are hard to differentiate from their original cultivar based on a limited number of molecular markers^16^. Moreover, considering the high genetic similarity level, as indicated by the propagation characteristics of Chinese jujube, the redundant accessions with distinct phenotypes are suitable for functional genomic studies. For example, ‘Hupingzao,’ which is identical to ‘Junzao’ based on the SSR genotype (Table S2), is a selected cultivar from ‘Junzao’ with a different fruit shape. Other identical pairs identified in the study can be phenotypically differentiated based on various traits such as fruit size (e.g., ‘Zhanpudazao’ vs. ‘Xiaozao-C2’) and shape (e.g., ‘Lelingmopanzao’ vs. ‘Yuanling’). As Emanuelli *et al*.^16^ highlight, duplicate accessions with differing phenotypic traits could be especially valuable material for further studies of the regulation of important traits. Thus, it is necessary for the accessions with identical SSR genotypes to be further evaluated morphologically or to be investigated by more molecular markers before being considered for elimination from the collection.

The study evaluated the genetic diversity of a large Chinese jujube collection (622 unique diploid genotypes), representing the largest and most extensive study of this species to date. The number of alleles per locus (mean = 8.96) was much higher than that detected in 76 major Chinese jujube cultivars (mean = 5.70)^4^. The high level of allele variation may be due to the large number of accessions analyzed. The present study showed that the alleles are not evenly distributed in both repositories, partially due to the existence of many low frequency alleles (<0.05). This is verified by levels of 58.2% and 50.6% for Cangzhou and Taigu, respectively. Somatic mutation is important for the breeding of Chinese jujube, which has been kept by the vegetative method, producing an excess of low-frequency variants^20^. As a consequence, it is necessary to strengthen the protection of rare alleles, especially in the Cangzhou collection.

In accordance with our previous studies, which revealed the mean values of Ho (0.678) and He (0.621) using 31 SSRs^4^, high heterozygosity levels (Ho of 0.64 and He of 0.60) were detected in the present study. The results also agree with a recent report from an assessment of the entire genome sequence^9^. Several studies have shown that cash trees, such as *Citrus*^21^, *Diospyros kaki Thumb*^22^ and *Castanea crenata*^23^, also exhibited high genetic heterozygosity. The results may be explained by cross-pollination arising in fruit trees, including Chinese jujube, which are propagated vegetatively. It is necessary to plant different varieties together to ensure cross-pollination in order to overcome the prevalence of self- and cross-incompatibility. Other causes may be related to long-term natural selection, the mixed nature of the accessions or the historic mixing of strains from different populations^24^.

Structure and cluster analyses are effective means for studying genetic relationships related to germplasm resources^25^,^26^. *Structure* analysis showed that the grouping was largely consistent with the UPGMA clustering (Fig. 4). Considering the higher genetic diversity levels in groups 1 and 3, a higher percentage of mixed ancestry rate derived from the genotypes in group 2 may have occurred. The low proportion of the variance explained by the first two axes of the PCoA indicated that the planar graph may not efficiently represent a large number of variables. Similar results have been previously reported by Belaj *et al*.^27^ and Leigh *et al*.^28^. Despite the loss of geographical origin information, the cluster differentiation is evident. The first axis separated the majority of accessions in cluster 2 from those in clusters 1 and 3, whereas the second axis separated the majority of the accessions in cluster 1 from those in clusters 2 and 3. However, a small degree of admixture existed in the first two axes, suggesting that no strict distinction exists among the three clusters. The results can also be explained by the low molecular variance among the clusters (0.85%), indicating limited differentiation.

The taxonomic controversy between sour jujube and Chinese jujube is worth noting^29^. It was long considered that jujube was domesticated from wild jujube^30^,^31^,^32^. Some have classified Chinese jujube and sour jujube as two independent species based on the morphology, habitat, anatomy, and other differences^33^. Others have treated sour jujube as a subspecies based on a SRAP analysis and ITS sequence data^34^. However, these studies failed to make convincing arguments to defend their positions. In the present study, sour jujube accessions were clustered into two different clades (Fig. 4). *Structure* analysis also divided the sour jujube accessions into two different groups, indicating that different groups may have been independently domesticated, which agrees with the results of a cpDNA analysis^35^. Thus, the present study supports the view that sour jujube should not be recognized as a unique species.

The major consideration in constructing a core collection from a very large germplasm collection is to develop reliable classification criteria. However, the problem does not exist within Chinese jujube accessions. Groups of accessions defined by the growing region, cultivar origin, species or subspecies proposed in previous studies cannot be effectively applied to Chinese jujube^15^,^36^. Consequently, a non-group strategy is adopted to construct a core collection. Previous studies have proposed differing criteria regarding the relative sizes of core collections^37^,^38^,^39^,^40^. Most researchers believe that 5–20% of the sampled size should encompass the genetic diversity of the entire collection. In the present study, we designed a large scale core set (2–40% of the 947 diploid accessions). A comparison of the sampling efficiency (i.e., the ability to capture allele numbers) supports the simulated annealing algorithm as the favorable approach (Fig. 5).

Large plant collections are expensive to maintain. Thus, a minimum number of samples that represent maximum genetic diversity is recommended^41^. The results suggest that the subset with a 15.6% sampling ratio yielded the largest allelic retention (99.5%). Similar studies have reported allelic retention values of 95.74% and 100% in pear^42^ and melon^17^, with sampling ratios reaching 24.2% and 19.4%, respectively. Only 4% of the sampling proportion sufficiently captured the entire genetic diversity of analyzed grapevine collections, which may be due to the high heterozygosity level and redundancy in the collection^43^.

Twenty accessions listed in Table S5 were retained to assure representation of the characteristics in the core collection. The retained accessions, representing each of the unique phenotypic characteristics not included in the core, could easily be added to the core collection. Moreover, the complicated relationships shown in Fig. 4 reflect the admixture within Chinese jujube, and pre-selection can avoid rejection of high-quality accessions. Larger subsets analyzed in the study guarantee full allelic coverage and maximum genetic diversity, especially considering the abundance of low-frequency alleles (59.5%). Different core collection strategies have been reported^15^,^17^,^36^,^37^,^42^. The simulated annealing algorithm implemented in PowerMarker software ensured a high allelic coverage, while PowerCore software yielded maximum allele diversity with the lowest sampling intensity. The combination of the two strategies is useful for conservation purposes. No significant difference was observed in the variability parameters and allele frequency distribution between the core and entire unique collections, indicating that the core collection developed in the present study effectively represents the 622 unique genotypes. Due to the lack of a proper characterization method and/or a large number of germplasms, reliable data related to important plant traits, such as the disease tolerance (Witches Broom, fruit shrink disease, etc.), freezing, fruit cracking, fruit quality, and other factors, may not be available. The genetic marker data are limited, and morphological diversity may be lost if they are used solely to determine the core collection. Therefore, it is crucial to characterize the accessions morphologically. The development of a core collection can facilitate the enhanced characterization of important jujube traits.

A valuable core collection should be dynamic and periodically revised to incorporate additional accessions^44^. Furthermore, we can determine new core collections that are suitable for other users. Core collections can provide a rational framework for intensive natural variation surveys linked to complex traits, such as fruit cracking resistance in Chinese jujube, which can improve utilization and breeding.

## Methods

### Plant material and genomic DNA extraction

In total, 962 *Ziziphus* accessions were collected from the National Chinese Jujube Germplasm Repository located in Taigu County, Shanxi Province (Taigu) and the National Foundation for Improved Cultivar of Chinese Jujube, Cang County, Hebei Province (Cangzhou) (Table S6). The samples consist of 942 diploid accessions and 15 triploid accessions of *Z. jujuba* var. jujuba, as well as five accessions of sour jujube (Table S7). All collected fresh young leaves for each accessions were immediately flash frozen in liquid nitrogen and stored at −70 °C until use. Total genomic DNA was extracted from the leaves following described methods^4^. DNA quality was tested using 1.0% agarose gel electrophoresis, and the DNA was diluted to 10 ng/μL.

### SSR markers and PCR reaction

A set of 24 SSR loci scattered throughout the genome were selected on the basis of their polymorphism and reproducibility^4^ (Table 2). PCR amplifications were performed on 10 μL volumes containing 1 μL of template DNA (10 ng/μL), 5 μL of 2x Taq mix, 0.4 μL of the forward primer (1 μM), 1.6 μL of the reverse primer (1 μM), 1.6 μL of M13 primer (1 μM) with a fluorescent label (FAM, HEX, ROX, or TAMRA), and 0.4 μL of ddH2O. The thermal cycling program consisted of pre-denaturation at 94 °C for 5 min, 30 cycles at 94 °C for 30 s, 55 °C for 30 s, and 72 °C for 40 s, followed by 8 cycles at 94 °C for 30 s, 53 °C for 30 s, 72 °C for 40 s, and a final extension at 72 °C for 10 min^45^.

PCR products were analyzed via capillary electrophoresis using an ABI 3730XL DNA Sequencer (Applied Biosystems, Foster City, CA, USA). Alleles were identified using the GeneMarker v 1.75 software package (SoftGenetics LLC, State College, PA, USA).

### Genetic diversity analysis

Microsatellite alleles were corrected using FlexiBin v 2^46^ and GeneMarker v 1.75 (SoftGenetics LLC, USA). The Microsatellite toolkit v 3.1.1^47^ was used to identify the duplicate sets. The remaining accessions with unique SSR genotypes were used to estimate the following parameters using GenAlEx 6.5^48^: the number of alleles (Na), the effective number of alleles (Ne), observed heterozygosity (Ho), expected heterozygosity (He), the probability of identity (PI), and polymorphic information content (PIC).

### Population structure analysis

A Bayesian clustering analysis was implemented in *Structure* 2.3.3^49^,^50^ to evaluate population genetic structure. An admixture model and correlated allele frequencies were applied to estimate the ancestry fractions of each cluster attributed to each accession. For each value of K (range 1–20), twenty independent runs were performed with a burn-in period of 200,000 followed by 500,000 MCMC (Markov chain Monte Carlo) repetitions. Parameters were set to the default values, and all accessions were treated as having unknown origins. The delta K method^51^ was implemented in Structure Harvester^52^ to determine the most probable value of K. The accessions with membership probabilities greater than or equal to 0.50 were considered to belong to the same group. Diversity statistics were calculated in PowerMarker v 3.25^53^ based on the genetic clusters identified by *Structure*, including the genotype numbers of each cluster, major allele frequency, number of alleles, genetic diversity, and polymorphic information content. An unweighted pair group method with an arithmetic mean (UPGMA) dendrogram was constructed using PowerMarker v 3.25^53^. A principal coordinate analysis (PCoA), based on the standardized covariance of genetic distances was performed using GenAlEx v 6.5^48^.

### Core collection development

Given the absence of detailed genetic information about the accessions, a non-group-based strategy was adopted. Four steps were executed: (1) Twenty-one core collections corresponding to different scales were constructed to identify the optimal core collection size (the sampling scale increased from 20 to 400 in increments of 5,10, 50, and 100 when the scale ranging from 20 to 50, 50 to 150, 150 to 300, and 300 to 400, respectively). Five independent runs were then repeatedly performed for each core selection by using two algorithms, including simulated annealing algorithm and random algorithm, implemented in PowerMarker to determine the optimal core collection size. (2) Several accessions that are agronomically (cracking resistance, fruit size, etc.) and commercially important (e.g., ‘Huanghuadongzao,’ ‘Xinzhenghuizao’ and ‘Junzao’) were selected as the retained accessions (listed in Table S5). (3) PowerMarker software^53^ and PowerCore software^54^ selected accessions based on the allele number and genetic diversity. The PowerCore software used a heuristic algorithm. A total of 1,000 independent runs were conducted based on the PowerMarker software^54^. Accessions with occurrence numbers above 500 were then retained in the core collection. The results from the analysis by the two softwares were combined for further screening. (4) Based on the dendrogram, one of the accessions with a close relationship was removed until the optimal core size was reached.

SPSS v18.0 (SPSS, Chicago, IL, USA) was used to assess the final core collection by performing Levene’s test and T-test for Na, Ne, Ho, He, and PIC values between the core and the entire unique collection. The comparison of allele frequency were carried out with Microsoft Excel (Microsoft, Washington, USA).

## Additional Information

**How to cite this article**: Xu, C. *et al*. Identifying the genetic diversity, genetic structure and a core collection of *Ziziphus jujuba* Mill. var. jujuba accessions using microsatellite markers. *Sci. Rep.* **6**, 31503; doi: 10.1038/srep31503 (2016).

## Supplementary Material

Supplementary Information

## Figures and Tables

**Table 1:** List of diploid accessions information and genetic parameters for Cangzhou and Taigu.

Category	CangZhou	Taigu	Total
Accessions analyzed	479	468	947
Accessions with one or more Private Alleles	57	23	80
Number of distinct SSR genotypes	362	315	622
SSR genotypes represented by one accession	281	218	499
SSR genotypes represented by two or more accessions	81	97	123
Number of Alleles	196	176	215
Number of Private Alleles	39	19	—
Mean Different Alleles	8.17	7.33	8.96
Mean Effective Alleles	2.93	3.03	3.03
Mean Observed Heterozygosity	0.62	0.64	0.64
Mean Expected Heterozygosity	0.58	0.60	0.60
Mean Polymorphic Information Content	0.53	0.56	0.55

**Table 2:** Observed probability of identity calculated from 622 unique genotypes using GenAlEx 6.5 software on 24 SSR loci.

SSR Marker	Number of identical pairs of genotypes	Probability of identity	Cumulative probability of identity
BFU0308	6517	2.3E-02	2.3E-02
BFU1157	1521	6.1E-02	1.4E-03
BFU0574	821	7.4E-02	1.0E-04
BFU1205	676	8.5E-02	8.7E-06
BFU0467	589	8.6E-02	7.4E-07
BFU0377	356	1.0E-01	7.5E-08
BFU0286	240	1.1E-01	8.2E-09
BFU1409	139	1.2E-01	1.0E-09
BFU0586	133	1.3E-01	1.3E-10
BFU1178	123	1.5E-01	1.9E-11
BFU0277	114	1.7E-01	3.3E-12
BFU1279	100	1.9E-01	6.3E-13
BFU0733	98	1.9E-01	1.2E-13
BFU0539	98	2.1E-01	2.5E-14
BFU0564	90	2.2E-01	5.5E-15
BFU0479	44	2.4E-01	1.3E-15
BFU0478	12	3.3E-01	4.4E-16
BFU0263	8	3.4E-01	1.5E-16
BFU0501	8	3.5E-01	5.1E-17
BFU0249	8	3.7E-01	1.9E-17
BFU0473	8	4.2E-01	8.0E-18
BFU0614	4	4.2E-01	3.4E-18
BFU0584	3	4.5E-01	1.5E-18
BFU0521	0	5.6E-01	8.6E-19

**Table 3:** Genetic diversity statistics for 24 SSR loci in 622 unique genotypes.

Locus	Na	Ne	Ho	He	F(Null)	PIC
BFU0263	5	2.04	0.7	0.51	−0.37	0.41
BFU0478	4	1.86	0.5	0.46	−0.1	0.42
BFU1205	7	4.51	0.83	0.78	−0.07	0.74
BFU0586	11	3.41	0.82	0.71	−0.15	0.66
BFU0377	14	4.08	0.8	0.76	−0.05	0.71
BFU0539	13	2.64	0.73	0.62	−0.18	0.56
BFU1279	6	2.54	0.56	0.61	0.073	0.57
BFU0249	6	1.97	0.48	0.49	0.025	0.38
BFU0733	3	2.92	0.76	0.66	−0.152	0.58
BFU0467	12	4.42	0.89	0.77	−0.155	0.74
BFU0308	21	8.97	0.91	0.89	−0.024	0.88
BFU0473	6	1.63	0.42	0.39	−0.074	0.34
BFU0584	3	1.62	0.4	0.38	−0.044	0.31
BFU1157	14	5.28	0.86	0.81	−0.059	0.79
BFU0501	4	1.82	0.5	0.45	−0.102	0.4
BFU0614	6	1.71	0.39	0.42	0.061	0.34
BFU1178	5	3.3	0.74	0.7	−0.061	0.64
BFU0479	14	2.23	0.25	0.55	0.55	0.51
BFU0574	13	4.32	0.82	0.77	−0.065	0.75
BFU0521	10	1.35	0.26	0.26	0.009	0.25
BFU0286	9	3.9	0.65	0.74	0.122	0.7
BFU1409	13	3.53	0.69	0.72	0.033	0.67
BFU0564	7	2.64	0.74	0.62	−0.2	0.54
BFU0277	9	2.98	0.67	0.66	−0.012	0.61
Mean	8.96	3.15	0.64	0.61		0.56

Na: Numbers of alleles; Ne: Number of Effective Alleles; HO: Observed heterozygosity; HE: Expected heterozygosity; F(null): Null alleles; PIC: Polymorphic Information Content.

**Table 4:** Comparison of the genetic diversity and significance test of the differences between the 622 unique diploid genotypes collection (215 alleles) and core collection (214 alleles).

	Mean value of the entire collection	Mean value of the core collection	Levene’s test	T-test
Na	8.96	8.92	0.66	0.59
Ne	3.15	3.42	0.9	0.46
Ho	0.64	0.64	0.75	0.55
He	0.61	0.64	0.66	0.97
PIC	0.56	0.59	0.8	0.54

Levene’s test: Levene’s test for equality of variances; T-test: T-test for equality of means.
