# Supplementary material for: Identifying the genetic diversity, genetic structure and a core collection of Ziziphus jujuba Mill. var. jujuba accessions using microsatellite markers
Source: Sci Rep. 2016 Aug 17;6:31503. doi: 10.1038/srep31503 (PMC4987672; doi:10.1038/srep31503)
Supplement: Supplementary Information [file srep31503-s1.pdf]

Identifying the genetic diversity, genetic structure and a core collection of *Ziziphus jujuba* Mill. var. jujuba accessions using microsatellite markers

Chaoqun Xu[1], Jiao Gao[1], Zenfeng Du[2], Dengke Li[3], Zhe Wang[1], Yingyue Li[1], Xiaoming Pang[1]*

Table S1 List of the 622 SSR genotypes

SSR genotypes represented by one accession (499 accessions)			SSR genotypes represented by two or more accessions(123 accessions)		
Code	Accession name	Preserved location	Code	Accession name	Preserved location
1	Dalilongzao-C	C	4	Wuhezao-C1	C
2	Gutouxiaozao	C	5	Lizao	C
3	Yuanlingxiaozao	C	6	Dongzao-103	C
13	C50	C	7	Zaocuiwang	C
14	Huairoudacuizao	C	8	Pozao-C1	C
15	S182	C	9	Wuhezao-C2	C
16	Langjiayuanzao-C	C	11	Mayizao-C	C
17	Wuhezao-C3	C	12	Yiwuzao	C
18	Shaanxigedazao	C	21	Manmanzao	C
19	Jidanzao-C1	C	28	Popozao	C
20	Sanlengzao	C	37	Chahuzao-C	C
23	C02	C	39	Shengxianbaipuzao-C	C
24	C03	C	45	Goutouzao	C
26	C52	C	46	Dalingzao	C
30	Longxuzao	C	49	Wutouzao	C
31	Dongzao-70	C	50	C06	C
33	C01	C	52	Jianzao-C1	C
36	Chaoyangyuanzao	C	56	Wuhezao-C5	C
38	Lianxianmuzao-C1	C	59	Huluchanghong-C	C
40	Xuezao	C	62	Shanghaibaipuzao-C	C
41	Lengzao-224	C	63	Ruanhezao-C1	C
42	Mingshandazao	C	74	Xiangzao-45	C
43	Xiaoyuanzao	C	75	Malianxiaozao	C
44	C05	C	76	Chuanlingzao-C1	C
47	Damuzao	C	77	Wuhezao-C6	C
51	Henan-4	C	81	Dunhuangdazao-C	C
53	Banzao-C	C	82	Dayewuhezao	C
54	Jinzao-C1	C	95	Fengmiguan	C
55	Xiaozizao	C	99	Wuhezao-C7	C
58	Duanguochanghong	C	107	Mayazao	C
60	Xuanchengyuanzao	C	110	Bashenghu	C
61	C07	C	121	Linglingzao	C
64	Jiebuqi	C	124	Songxiandazao-C	C
65	Rodouzizao	C	127	Zhentouzao	C
66	C08	C	139	Meimizao	C
67	C09	C	148	Chengwudongzao-T	C
69	Dasuanzao	C	149	Liuyuexian-C	C
71	Dongzao-40	C	154	Tailihong	C
72	Lingbaodazao-C	C	171	Xiaozao-C21	C
73	Xingguang	C	172	Mamazao-C1	C
78	Henan-6	C	173	Gedazao-C1	C
79	Henan-7	C	174	Suyuanling-C	C
80	C10	C	191	Xiaozao-C27	C

84	Wanmianzao	C	196	Kongfucui	C
85	C12	C	198	Jinai-NO.2	C
86	Mianxiaozao	C	201	Lengbaiyu	C
87	Ao-3	C	217	Sanbianchou-C	C
89	Jinai-NO.4	C	218	C19	C
90	Lichengxiaozao-C	C	229	Dabailing	C
91	Mayazao-C2	C	234	Daguazao-C	C
92	Dongzao-38	C	240	Luzao-NO.11-C1	C
93	Xiangfenmuzao-C	C	244	Luzao-NO.9-C1	C
94	Tuanzao	C	255	Jinsi-NO.4-C	C
96	Hamazao	C	274	Beijinghuluzao-C	C
97	Lajiaozao-C1	C	275	Jiaxianchangzao	C
98	Zizao-C1	C	278	Heigeda	C
101	Zaoshutangzao	C	280	Tengzhouchanghong-C	C
104	Wanshuyuanling	C	284	Tianjinkuaizao	C
105	Xinmopan	C	288	Yiwuezizao	C
106	S161	C	301	C36	C
109	Niujiaozao	C	304	Hubeiyuanzao	C
111	Cuizaohong	C	308	Baodeyouzao-C	C
112	Yuanlizao-C1	C	311	C39	C
113	C13	C	315	Hongzhaoshiyuehong-C	C
114	Xiaozao-C3	C	331	Kulianzao-C	C
115	Jinmangguo	C	332	Xiaoguosuanpanzao-C	C
116	Suanjixinzao	C	336	Dingxiangxingxingzao-C	C
118	Xiaozao-C5	C	343	Lengzao-C	C
122	Xiaozao-C8	C	348	Muzao-C	C
123	Xiaozao-C9	C	350	Yanliangxiangzao	C
125	Guangyangdazao	C	353	Manaizao	C
126	Yueyazao	C	374	BJ15	C
128	Jinsixin-NO.1	C	389	BJ32	C
129	Mopanzao-C	C	397	BJ48	C
130	Gagazao-C	C	404	BJ34	C
131	Guantanzao	C	412	BJ50	C
132	Wenxianshatang	C	457	Jidanzao-C3	C
133	C14	C	465	Henan-9	C
136	Wuhezao-C8	C	472	Lincui-NO.1	C
137	Xiaozao-C10	C	477	Hongzhaohuluzao-C	C
138	Buluosu	C	481	Yuanquzao-C	C
140	C15	C	492	Cangxiantunzizao	T
141	Jidanzao-C2	C	493	Cangxianxiaozao	T
142	Tangzao-C1	C	494	Shulutangzao	T
143	Shazao	C	498	Shenxianchuanganhong	T
144	Xiaozao-C11	C	517	Qingyuandadanzao	T
145	Xiaozao-C12	C	519	Liyiwumingzao	T
146	Henan-12	C	522	Jinnan-NO.1	T
147	Yingbuluo	C	531	Xianxiansuanzao	T
150	Maoertou	C	547	Xinzhengjixinzao	T
152	Mianzao	C	561	Yucinaitouzao	T

155	Xiaomuzao	C	563	Yucichangmuzao	T
157	Linfenmizao-C	C	584	Guantan-NO.1	T
159	Manchengtuntunzao	C	589	Dalimayazao	T
160	Duanzizao-C	C	593	Puchengdundunzao	T
161	Tangzao-C	C	648	Midiancuimuzao	T
162	Daxuezao	C	690	Laopozao-T	T
167	Xiaozao-C17	C	695	Langjiayuanzao-T	T
169	Xiaozao-C19	C	705	Qiyuexian	T
170	Xiaozao-C20	C	710	Changdazao	T
175	Zhongyangmuzao-C	C	750	Dahongzao	T
177	Gansuxiaokou	C	770	Wukuzhakexiaozao	T
180	Cuzao	C	772	Kuerlexiaozao	T
181	S181	C	778	Yixianmuzao	T
185	Xiaozao-C22	C	787	Dingxiangxiaozao	T
194	C17	C	790	Wutaimianzao-T1	T
199	C18	C	795	Malingsuan	T
204	Xiaozao-C28	C	804	Habazao-T	T
208	Xiaozao-C32	C	806	Shouxingzao	T
210	Xiaozao-C34	C	808	Daling	T
211	Xiaozao-C35	C	820	Jiaochengyazao	T
214	Xiaozao-C38	C	832	Taigulonghuzao	T
215	Xiaozao-C39	C	843	Linfenmizao-T	T
219	C20	C	845	Linfentuanzao	T
220	Huizao	C	850	Hongzhaocuizao	T
222	Yazao-C176	C	891	Huxiantaipingjianzao	T
223	Budaizao	C	900	Zhumadianyangjiaozao	T
224	Shanxitedage	C	909	Wutaimianzao-T2	T
225	Beijingmayazao-C	C	911	Jingzao-39	T
227	Jinai-NO.1	C	921	Beijingpaopaozao	T
230	C22	C	922	Changxindianbaizao-2	T
235	Luzao-NO.8-C1	C	925	Baozhuangxiaoyuanzao	T
237	C25	C	946	Chaoyangxiaopingding	T
238	Hengshuibianzhi	C			
242	Luzao-NO.10-C1	C			
243	Luzao-NO.1	C			
245	Shishengmopan	C			
247	Yueyacui	C			
249	Luzao-NO.7	C			
251	Luzao-NO.6	C			
253	Suizhoudazao	C			
254	Zhongqiusucui	C			
260	Muzaokanglie-NO.1	C			
262	BJ11	C			
264	BJ21	C			
265	C29	C			
270	Sanbianhong	C			
273	Laohuyan	C			
276	C32	C			

277	Shandonglongzao	C			
279	C33	C			
281	Dalishuizao	C			
282	Jinchang-NO.2	C			
283	Beibeixiaozao-C	C			
285	Qianlingdazao-C	C			
286	Yingluozao-C	C			
289	Pinglubangchuizao	C			
290	Xinzhengzaohong	C			
291	Jikang	C			
293	Jinzao-NO.3	C			
294	Yuanlizao-C2	C			
295	Linyuzao	C			
296	Xiaosicui	C			
297	C34	C			
298	Tengzhoudamaya	C			
299	Hongzhaoxiaozao-C	C			
300	C35	C			
303	Guangyang-NO.2	C			
305	Xishuangxiaozao-C	C			
306	C38	C			
309	Youhezao-C	C			
310	Fengyuancui	C			
312	Yunnan-NO.2-C	C			
313	Lincui-NO.2	C			
314	Xuyiyanlaihong	C			
316	Xuanchengjianzao-C	C			
317	Shaanxixiaodundunzao	C			
318	Fucuihong	C			
319	Xiyaoyaozaozao	C			
321	Habazao-C	C			
322	Hejinjinzao-C	C			
323	Akesuxiaozao-C	C			
324	Dalilinglingzao-C	C			
325	C40	C			
326	Zhenhuluzao	C			
327	Taiguduanzizao-C	C			
328	Shanxigedazao	C			
329	Zhongyangtuanzao-C	C			
330	Lianxianmuzao-C2	C			
333	C41	C			
335	C43	C			
338	Luzao-NO.2	C			
339	Luzao-NO.10-C2	C			
341	Luzao-NO.8-C2	C			
344	Jinxuan-NO.1	C			
347	Yanliangcuizao	C			
351	Jingzao-60	C			

352	Miguanxin-NO.1	C			
354	Fuzao-C	C			
356	Yingshanhong	C			
357	Dongzao	C			
358	Wanronggedazao	C			
360	Wuxiangtianzao	C			
361	C44	C			
362	Hunanchangzao	C			
363	Linyibenzao-C	C			
364	BJ22	C			
365	BJ12	C			
366	BJ23	C			
367	BJ26	C			
368	BJ24	C			
369	BJ19	C			
370	BJ20	C			
371	BJ25	C			
372	BJ27	C			
373	BJ01	C			
375	BJ16	C			
377	BJ08	C			
378	BJ05	C			
379	BJ06	C			
380	BJ02	C			
381	BJ03	C			
382	BJ04	C			
383	BJ09	C			
384	BJ07	C			
385	BJ10	C			
386	BJ13	C			
391	BJ36	C			
392	BJ37	C			
393	BJ40	C			
394	BJ41	C			
395	BJ44	C			
398	BJ49	C			
399	BJ52	C			
400	BJ53	C			
402	BJ28	C			
403	BJ31	C			
406	BJ38	C			
410	BJ46	C			
416	C45	C			
417	Daguoshengzhouchang	C			
418	Yuexiangcuizao	C			
419	Yuanguoshengzhoubai	C			
420	Zidantou	C			
421	Xiaoguoshengzhouyuan	C			

422	Qiguzao	C			
423	Niunaizao	C			
424	Yejiatianzao	C			
427	C46	C			
429	Jianzao-C2	C			
430	BJ57	C			
431	BJ14	C			
432	Aowang	C			
433	Fupingdazao	C			
434	Xiaopingding	C			
435	Lucui	C			
437	Xuanlingzao-C	C			
438	Bopicui	C			
439	C47	C			
441	Maguzao	C			
442	ZhongguoShengzhouch	C			
444	Liuyuehong	C			
446	Changzicui	C			
447	Hunanlongxuzao	C			
448	Hunantailihong	C			
452	Lingzao-C	C			
453	Daguoshengzhouyuanz	C			
456	Junzao	C			
458	Jiangchuang-NO.1	C			
459	Jikang-NO.1	C			
460	Changhong	C			
461	Jixinzao-C	C			
463	Lajiaozao-C2	C			
464	Xiaozao-C41	C			
467	C49	C			
468	Mamazao-C2	C			
469	Wuhezao-C10	C			
470	Longzao	C			
471	Jinzao-C2	C			
473	Suancuiwang	C			
475	Kuduanzao	C			
476	Xupuliuyuezao	C			
478	Suanzao	C			
479	Dongzaoerdai	C			
480	Yanchuanbaizao-C	C			
482	Lincui-NO.5	C			
483	Shaanximianzao-C	C			
484	Zhishegedazao-C	C			
485	Taiguhuluzao-C	C			
486	Xinjiangwumingzao	C			
487	Tianzao	C			
488	Shaanxiqiyuexian	T			
489	Taigushenglizao	T			

490	Lichengchuanlingzao	T			
491	Xinledazao	T			
497	Jinxianmuzao	T			
500	Linyilajiao	T			
502	Xianxianlajiaozao	T			
507	Hebei-NO.13	T			
508	Xuechengdongzao	T			
512	Zaoqiangcuizao	T			
514	Yunchengmianzao	T			
515	Yunchenghamazao	T			
516	Yunchengcuizao	T			
520	Zaozhuanggongzao	T			
524	Zaoqiangshazao	T			
525	Jinsitedazao	T			
526	Xianxianmianzao	T			
527	Hanguoyuechu	T			
528	Hanguowudeng	T			
534	Guoyanhong	T			
535	Taigudasuanzao	T			
541	Xinzhengjidanzao	T			
544	Shandonglizao	T			
545	Beijingjidanzao	T			
552	Lingbaodazao-T	T			
553	Zhenpingguangyangzao	T			
554	Anyangtuanzao	T			
558	Huanghuadongzao	T			
560	Xinzhengdamaya	T			
562	Yuciwanhongzao	T			
566	Yuciyazao	T			
568	Xinzhengqitoubai	T			
570	Xinzhengxiaoyuanzao	T			
572	Puyangtangzao	T			
573	Linyilizao	T			
574	Yanchuandieyazao	T			
582	Lejin-NO.2	T			
585	Yongchengchanghong	T			
588	Dalixiaodundunzao	T			
590	Dalijidanzao	T			
591	Puchengmianzao	T			
592	Puchengyuanlizao	T			
594	Shaanximianzao-T	T			
597	Dalilongzao-T	T			
602	Jinzao-NO.1	T			
603	Dalipachizao	T			
604	Binxianyuanzao	T			
608	Binxianshuizao	T			
610	Puchengzhishezao	T			
616	Daligedazao	T			

620	Shaanxinaizao	T			
621	Yanchuanbaizao-T	T			
622	Dabaizao	T			
630	Liaochengyuanlingzao	T			
633	Shishengdongzao	T			
634	Dacuizao	T			
639	Lingzao-T	T			
640	Huluchanghong-T	T			
641	Sanbianse	T			
642	Jiuyuexian	T			
645	Xuanlingzao-T	T			
646	Shaanxixiaoyuanzao	T			
647	Huluzao	T			
649	Linqinzao	T			
650	Xiyaozao	T			
651	Yangnaizao	T			
652	Jiaxianbaizao	T			
653	Heyanglinglingzao	T			
655	Zheshegedazao-T	T			
656	Puchengjinzao	T			
659	Henanxiaozao	T			
660	Lingbaoling-NO.1	T			
661	Guoxingshibingzhuang	T			
664	Malingzao	T			
665	Lajiaozao-T	T			
668	Mopanzao-T	T			
670	Luodihong	T			
674	Houtouzao	T			
678	Lushibingzao	T			
679	Beibeixiaozao-T	T			
681	Guchengdongzao	T			
682	Yucituanzao	T			
686	Zilingdan	T			
689	Leng-3	T			
697	Yingluozao-T	T			
698	Jingxixiaobaizao	T			
704	Erqiuzao	T			
707	Kangtouzao	T			
708	Zhenzhuzao	T			
709	Xiangjianzao	T			
711	Tangzao-T	T			
714	Tiansuanzao	T			
715	Ruchengzao	T			
716	Daguosuanpanzao	T			
717	Chengtuozao-T	T			
718	Guanyinzao	T			
719	Xiangyuanzao	T			
720	Xiangmuzao	T			

721	Xiangzao	T			
722	Yanzao	T			
724	Chengchuizao	T			
727	Binlangzao	T			
729	Xupushibingzao	T			
731	Shatangzao	T			
732	Bopizao	T			
734	Shengxianbaipuzao-T	T			
735	Nanjingdamuzao-T	T			
736	Ezizao	T			
737	Suantianzao	T			
738	Mianxuzao	T			
739	Yiwudazao	T			
740	Mazao	T			
742	Shaizao	T			
749	Gansudongzao	T			
751	Diaolingzao	T			
755	Shuituanzao	T			
758	Yanlaihong	T			
760	Guanyangduanzao	T			
761	Guanyangchangzao	T			
762	Diaodiaopo	T			
767	Minqinxiaozao	T			
768	Ningxiachangzao	T			
769	Kashixiaozao	T			
771	Xinjiangxiaoyuanzao	T			
773	Changyuanzao	T			
775	Yuancuizao	T			
776	Akesuxiaozao-T	T			
777	Zunyitianzao	T			
779	Xishuangxiaozao-T	T			
780	Yunnan-NO.2-T	T			
781	Kunmingzao	T			
782	Kulianzao-T	T			
783	Guangdongbaizao	T			
784	Guangdongdazao	T			
785	Guangdongmuzao	T			
786	Guangdongtangzao	T			
789	Youhezao-T	T			
791	Yucijiuyueqing	T			
793	Maohouzao	T			
794	Qiutuanzao	T			
797	Hejinjinzao-T	T			
798	Taiyuanyuanzao	T			
799	Taiyuanchangzao	T			
800	Lvfendan	T			
802	Yazao	T			
807	Mugedazao	T			

811	Hejinshuizao	T			
818	Bolicui	T			
821	2001-1	T			
822	2001-2	T			
823	2001-3	T			
824	2001-5	T			
825	2001-4	T			
826	2003-1	T			
828	Damozao	T			
829	Linyizhenzhulongzao	T			
835	Yunchengxiangzao	T			
836	Duanzhizao-T	T			
837	Cuitian-NO.1	T			
838	Jishanbanzao-T	T			
841	Xiaxianziyuanzao	T			
847	Xiangfenyuanzao	T			
851	Xiaxianyuancuizao	T			
852	Linfenzhenhulu	T			
855	Pingshunjunzao	T			
856	Taiguhupingsuan	T			
857	Zhongyangmuzao-T	T			
858	Taigumeimizao	T			
860	Taigujixinmizao	T			
861	Qingxuyuanzao-T	T			
864	Taiguheiyezao	T			
865	Lichengdamazao	T			
866	Taiguhuluzao-T	T			
868	Taigudundunzao	T			
870	Taiguduanzizao-T	T			
874	Pingyaokuduanzao	T			
877	Jiaochengduanzao	T			
878	Xiangfenmuzao-T	T			
879	Jiaochengtiansuanzao-T	T			
880	Wenshuishazao	T			
884	Liuguanzao	T			
885	Jishanchangzao	T			
886	Jishanyuanzao	T			
887	Shiloucuizao	T			
889	Lingzao-T1	T			
890	Zuiguifei	T			
891	Binxiansuzao	T			
899	Jinsi-NO.4-T	T			
902	Hanguofuzao	T			
903	Hanguojinxiu	T			
904	Hanguohongyan	T			
906	Jiangchuang-NO.2	T			
907	Wutaiwudizao	T			
908	Zhuluyouyouzao	T			

913	Beijinghuluzao-T	T			
914	Beijingdacuizao	T			
915	Beijinggagazao-T1	T			
918	Yuetanzao-T1	T			
919	Beijinghuashengzao	T			
925	Beijingheiyaozizao	T			
927	Beijingxiaomizao	T			
929	Beijingsuanzao	T			
930	Baozhuangdalingzao	T			
931	Beijingsuzao	T			
934	Beijinglaohuyan	T			
939	Huairoucuizao	T			
940	Chaoyangmazao	T			
942	Chaoyangmiantaozao	T			
943	Hongcunbaizao-2	T			
945	Chaoyangdapingding	T			
947	Chaoyangdajianding	T			
948	Chaoyangxiaojianding	T			
950	Chaoyangjinlingyuanza	T			
953	Chaoyangxiaoyuanling	T			
954	Chaoyangpingzizao	T			
956	Chaoyangchengtuozao	T			
959	Chaoyangmopanzao	T			
960	Jinximuzao	T			

Table S2 List of putative duplication sets detected in this study, including overall analysis and separate analysis of of Cangzhou and Taigu germplasm repository			
Putative duplication set			
Germplasm repository	SSR profile	Accession number	Accession name
	1	4	Wuhezao-C1
		241	C26
		258	Wuhezao-C9
	2	5	Lizao
		88	Shaanxijidanzao
		103	Xupujidanzao-C
		134	Mantouzao
		135	Hetaowen-C
		200	Jinai-NO.3
	3	6	Dongzao-103
		190	Dongzao-100
	4	7	Zaocuiwang
		35	C04
		70	Cuizao-37
		102	Xiaolizao
		259	C28
	5	8	Pozao-C1
		151	Pozao-C2
	6	9	Wuhezao-C2
		32	Wuhezao-C4
		34	Jinsixiaozao-C1
		48	Jinsixiaozao-C2
		117	Xiaozao-C4
		120	Xiaozao-C7
		165	Xiaozao-C15
		166	Xiaozao-C16
		195	Changmuzao-C
		213	Xiaozao-C37
		250	Laopozao-C
		256	Beijingyingbuluo
		263	BJ18
		440	Tanzao-C
	7	21	Manmanzao
		22	C51
		25	Hupingzao-C1
		27	Hupingzao-C2
		425	Jinchang-NO.1-C
		462	Hupingzao-C2
	8	28	Popozao
		156	Qingxuyuanzao-C
	9	45	Goutouzao

	[illegible]	178	Bianshizao
	10	49	Wutouzao
		83	C11
	11	50	C06
		158	Heiyezao
	12	56	Wuhezao-C5
		57	Xiaozao-C1
		68	Yuanzao-206
		163	Xiaozao-C13
		164	Xiaozao-C14
		168	Xiaozao-C18
		182	S192
		186	Xiaozao-C23
		187	Xiaozao-C24
		188	Xiaozao-C25
		189	Xiaozao-C26
		192	Guazao-C1
		197	Jinsixin-NO.2
		205	Xiaozao-C29
		207	Xiaozao-C31
		209	Xiaozao-C33
		212	Xiaozao-C36
		228	Wuhe-NO.1
		236	C24
		239	Luzao-NO.3
		257	Guazao-C2
		261	Wumingzao
		266	Jinlingyuanzao
		267	Fucuimi
		268	Baohulu
		269	C30
		271	C31
		272	Qitoubai
		302	C37
		307	Shaoguanbaizao
		320	Zhongmoubenzao
		355	Xiaoyazao
		390	BJ33
		396	BJ45
**Cangzhou**		408	BJ42
		445	Hunanwumingzao
		455	Dapingding
	13	63	Ruanhezao-C1
		426	Ruanhezao-C2
	14	74	Xiangzao-45
		349	Shanxilizao
	15	76	Chuanlingzao-C1
		153	Chuanlingzao-C2

		221	C21
	16	77	Wuhezao-C6
		119	Xiaozao-C6
	17	81	Dunhuangdazao-C
		176	Linzedazao-C
	18	99	Wuhezao-C7
		100	Xiaozao-C2
		216	Xiaozao-C40
		232	C23
		233	Zhanpudazao
	19	107	Mayazao
		108	S006
	20	121	Linglingzao
		183	Jishanbanzao-C
	21	127	Zhentouzao
		226	Henanlongzao-C
		231	Damayazao-C
	22	139	Meimizao
		388	BJ30
	23	149	Liuyuexian-C
		179	C16
	24	171	Xiaozao-C21
		206	Xiaozao-C30
		287	Wanrongfuzao
		387	BJ29
	25	174	Suyuanling-C
		345	Zizao-C2
	26	191	Xiaozao-C27
		248	Luzao-NO.4
	27	196	Kongfucui
		246	Gedazao-C2
	28	198	Jinai-NO.2
		359	Xuputiansuanzao
	29	229	Dabailing
		252	C27
	30	240	Luzao-NO.11-C1
		340	Luzao-NO.11-C2
	31	244	Luzao-NO.9-C1
		342	Luzao-NO.9-C2
	32	274	Beijinghuluzao-C
		411	BJ47
	33	275	Jiaxianchangzao
		292	Nanjingdamuzao-C
	34	284	Tianjinkuaizao
		346	Zhishezao
	35	301	C36
		451	Mifengzao-C
		311	C39

	36	334	C42
		337	Luzao-NO.5
		428	Mizao-C
		449	Xinxing
	37	374	BJ15
		376	BJ17
		401	BJ56
	38	389	BJ32
		415	BJ55
	39	397	BJ48
		413	BJ51
		454	Jingzao-40
	40	404	BJ34
		405	BJ35
		407	BJ39
		409	BJ43
		414	BJ54
	41	412	BJ50
		436	Shanxiwumingzao
	42	465	Henan-9
		466	C48
	43	472	Lincui-NO.1
		474	Jiaochengtiansuanzao-C
	1	518	Hetaowen-T
		600	Qianlingdazao-T
		700	Lizao-NO.1
		701	Tianjin-NO.1
		712	Xupujidanzao-T
	2	627	Lelingxiaozao
		628	Lelingwuhexiaozao
		629	Hebeiwuhezao
		636	Changmuzao-T
		696	Miyunxiaozao
		703	Minzao
		706	Kuaizao
		728	Liuyuezao
	3	746	Wanmutouzao
		754	Nanjingzao
	4	537	Taiguhupingzao
		539	Jiaochengjunzao
		576	Hupingzao-NO.2
		577	Hupingzao-NO.1
		756	Wangcun-NO.1
	5	733	Lanximazao
		744	Wanniunaizao
		745	Xuanchengjianzao-T
	6	540	Xinzhengsuzao
		557	Linxianwutouzao

		658	Jiuyueqing
	7	532	Taigumeixinhe
		533	Taigumeixinhong
	8	530	Cangxianjinsixiaozao
		605	Zaoqiangmalianxiaozao
	9	501	Hamidazao
		587	Yongchengyuanhong
		764	Linzedazao-T
		765	Dunhuangdazao-T
		766	Anningxiaozao
		813	Fuzao-T
		817	Cuizao
	10	598	Dalilinglingzao-T
		615	Dalifengmiguan
		618	Zhongcaobenzao
	11	601	Binxianjinzao
		662	Songxiandazao-T
	12	503	Hebeilongzao
		619	Henanlongzao-T
		625	Tengzhoutangzao
		643	Damayazao-T
	13	671	Ludamazao
		675	Malingcui
		691	Mamazao-T
		702	Gagazao-T
	14	614	SS1
		624	Lelingmopanzao
		631	Yuanling-NO.1
		632	Yuanling-NO.2
		635	Suyuanling-T
		694	Yuanling
	15	549	Puyangsanbianhong
		669	Sanbianchou-T
	16	546	Huizaozhiban-NO.1
		548	Xinzhenghuizao
		559	Xinzhengchangjixinzao
	17	676	Daguazao-T
		680	Fengjiejidanzao
	18	606	Binxianheigeda
		894	Binxianhonggeda
	19	612	Dalizhizao
		613	Xiyingbenzao
	20	657	Pozaozhibian-NO.1
		788	Shanzao
		863	Dingxiangxingxingzao-T
		550	Yucidamozao
		583	Yanchuantiaozao
		595	Lajiaozao-NO.1

	21	596	Lajiaozao-NO.2
		666	Wuxingzao
		667	Muzao-T
		677	Shidizao
		815	Hejintiaozao
		872	Pingyaodazao
		876	Xiangfenyazao
		910	Wutaimuzao
	22	687	Banzao-T
		688	Leng-2
		941	Hongcunbaizao
	23	492	Cangxiantunzizao
		495	Yutianxiaozao
		504	Xianxian-NO.21
		505	Xianxiandaxiaozao
		506	Xianxianmuzao
		510	Zaoqianggutouxiaozao
		511	Xianxianxiaoxiaozao
		521	Cangxianchangxiaozao
		529	Xianxianyuanxiaozao
		538	Gusuxiaozao
		567	Beijingbenzao
		569	Xinzhengdazao
		579	Lejin-NO.3
		580	Lelingwuhe-NO.2
	24	493	Cangxianxiaozao
		578	Lejin-NO.4
		581	Lejin-NO.1
	25	494	Shulutangzao
		556	Neihuangbianhesuan
		672	Chahuzao-T
	26	498	Shenxianchuanganhong
		536	Shenxianchuanganzao
		685	Chuanganzao
	27	517	Qingyuandadanzao
		551	Beijingzhuizibai
**Taigu**	28	519	Liyiwumingzao
		726	Changzao
		834	Yongjihamazao
	29	522	Jinnan-NO.1
		542	Xincaidayuanfeng
	30	531	Xianxiansuanzao
		713	Yuanzao
	31	547	Xinzhengjixinzao
		571	Xinzhengjiantouhuizao
	32	561	Yucinaitouzao
		565	Yucimianzao
		563	Yucichangmuzao

	33	699	08-1
		805	Bangchuizao
		831	Linhuang-NO.1
		842	Taigulajiao-NO.1
		844	Taigulajiao-NO.2
		854	Taigulangzao
		869	Pingshunbenzao
		882	Baodexiaozao
		897	Jiaxianchangmuzao
	34	584	Guantan-NO.1
		586	Guantan-NO.2
		684	Guantanwang
		849	Xiangfenguantanzao
	35	589	Dalimayazao
		599	Binxiansuangedazao
	36	593	Puchengdundunzao
		609	Daliyuanzao
		611	Daliganweiba
	37	648	Midiancuimuzao
		654	Shaanxiyazao
	38	690	Laopozao-T
		905	Baodingyueguang
		917	BeijinggagazaoT2
		923	Beijingmayazao-T
		932	Baozhuangjianzao
	39	695	Langjiayuanzao-T
		747	Jixinzao-T
	40	705	Qiyuexian
		803	Yongjijidanzao
	41	710	Changdazao
		723	Mizao-T
	42	750	Dahongzao
		752	Zhongningxiaozao
		763	Linzexiaozao
	43	770	Wukuzhakexiaozao
		816	Jinudazao
		819	Qingxumoguzao
		833	Jinchang-NO.1-T
		846	Taiguhuping-NO.1
		895	Fupingjidanzao
		898	Jinsi-NO.3-1
		944	Chaoyangwuhezao
		951	Chaoyangjinsimi
	44	772	Kuerlexiaozao
		938	Chaoyangqicuizao
	45	778	Yixianmuzao
		814	Linyibenzao-T
		787	Dingxiangxiaozao

	46	792	Mianmeizao
		801	Tanzao-T
		853	Lichengxiaozao-T
		928	Baozhuangxiaozao
		933	Beijingtaizimu
		937	Suziyumizao
		952	Chaoyangmeixinzao
		961	Jinsi-NO.3-2
	47	790	Wutaimianzao-T1
		859	Pingyaobuluosu
	48	795	Malingsuan
		796	Taiyuanshiyuehong
	49	804	Habazao-T
		810	Jidanzao-T
		812	Bobozao
	50	806	Shouxingzao
		883	Pinglutuntunzao
	51	808	Daling
		809	Xiaolingzao
		839	Pinglujianzao
	52	820	Jiaochengyazao
		862	Taigulinglingzao
	53	832	Taigulonghuzao
		848	Taiguhuping-NO.2
	54	843	Linfenmizao-T
		916	Beijing-31
	55	845	Linfentuanzao
		867	Zhongyangtuanzao-T
	56	850	Hongzhaocuizao
		871	Hongzaoxiaozao-T
	57	891	Huxiantaipingjianzao
		892	Huxiantaipingshuizao
		896	Lintongmalianzao
	58	900	Zhumadianyangjiaozao
		901	Tongbaidazao
	59	909	Wutaimianzao-T2
		912	Wutaicuzao
	60	911	Jingzao-39
		920	Yuetanzao-T2
		962	Xiangjingzao-39
	61	921	Beijingpaopaozao
		935	Changpingdahongpao
	62	922	Changxindianbaizao-2
		924	Changxindianbaizao-1
	63	926	Baozhuangxiaoyuanzao
		936	Chaoyangwanzao
		946	Chaoyangxiaopingding
		949	Chaoyangjinlingchangzao

	64	955	Chaoyanglingzao
		957	Ershijiadazao
		958	Gendedazao
	1	4	Wuhezao-C1
		241	C26
		258	Wuhezao-C9
	2	5	Lizao
		88	Shaanxijidanzao
		103	Xupujidanzao-C
		134	Mantouzao
		135	Hetaowen-C
		200	Jinai-NO.3
		518	Hetaowen-T
		600	Qianlingdazao-T
		700	Lizao-NO.1
		701	Tianjin-NO.1
		712	Xupujidanzao-T
	3	6	Dongzao-103
		190	Dongzao-100
	4	7	Zaocuiwang
		35	C04
		70	Cuizao-37
		102	Xiaolizao
		259	C28
		692	Qingyunxiaolizao
	5	8	Pozao-C1
		151	Pozao-C2
		509	Zaoqiangdongzao
	6	9	Wuhezao-C2
		32	Wuhezao-C4
		34	Jinsixiaozao-C1
		48	Jinsixiaozao-C2
		117	Xiaozao-C4
		120	Xiaozao-C7
		165	Xiaozao-C15
		166	Xiaozao-C16
		195	Changmuzao-C
		213	Xiaozao-C37
		250	Laopozao-C
		256	Beijingyingbuluo
		263	BJ18
		440	Tanzao-C
		627	Lelingxiaozao
		628	Lelingwuhexiaozao
		629	Hebeiwuhezao
		636	Changmuzao-T
		696	Miyunxiaozao
		703	Minzao

	706	Kuaizao
	728	Liuyuezao
7	11	Mayizao-C
	743	Mayizao-T
8	12	Yiwuzao
	746	Wanmutouzao
	754	Nanjingzao
9	21	Manmanzao
	22	C51
	25	Hupingzao-C1
	27	Hupingzao-C2
	425	Jinchang-NO.1-C
	462	Hupingzao-C3
	537	Taiguhupingzao
	539	Jiaochengjunzao
	576	Hupingzao-NO.2
	577	Hupingzao-NO.1
	756	Wangcun-NO.1
10	28	Popozao
	156	Qingxuyuanzao-C
	564	Yunchengpopozao
11	37	Chahuzao-C
	753	Nanjingyazao
12	39	Shengxianbaipuzao-C
	733	Lanximazao
	744	Wanniunaizao
	745	Xuanchengjianzao-T
13	45	Goutouzao
	178	Bianshizao
14	46	Dalingzao
	540	Xinzhengsuzao
	557	Linxianwutouzao
	658	Jiuyueqing
15	49	Wutouzao
	83	C11
16	50	C06
	158	Heiyezao
	532	Taigumeixinhe
	533	Taigumeixinhong
17	52	Jianzao-C1
	513	Linyiyazao
	56	Wuhezao-C5
	57	Xiaozao-C1
	68	Yuanzao-206
	163	Xiaozao-C13
	164	Xiaozao-C14
	168	Xiaozao-C18
	182	S192

	18	186	Xiaozao-C23
		187	Xiaozao-C24
		188	Xiaozao-C25
		189	Xiaozao-C26
		192	Guazao-C1
		197	Jinsixin-NO.2
		205	Xiaozao-C29
		207	Xiaozao-C31
		209	Xiaozao-C33
		212	Xiaozao-C36
		228	Wuhe-NO.1
		236	C24
		239	Luzao-NO.3
		257	Guazao-C2
		261	Wumingzao
		266	Jinlingyuanzao
		267	Fucuimi
		268	Baohulu
		269	C30
		271	C31
		272	Qitoubai
		302	C37
		307	Shaoguanbaizao
		320	Zhongmoubenzao
		355	Xiaoyazao
		390	BJ33
		396	BJ45
		408	BJ42
		445	Hunanwumingzao
		455	Dapingding
	19	59	Huluchanghong-C
		623	Yanchuanniunaicuizao
	20	62	Shanghaibaipuzao-C
		759	Shanghaibaipuzao-T
	21	63	Ruanhezao-C1
		426	Ruanhezao-C2
	22	74	Xiangzao-45
		349	Shanxilizao
	23	75	Malianxiaozao
		530	Cangxianjinsixiaozao
		605	Zaoqiangmalianxiaozao
	24	76	Chuanlingzao-C1
		153	Chuanlingzao-C2
		221	C21
	25	77	Wuhezao-C6
		119	Xiaozao-C6
		81	Dunhuangdazao-C
		176	Linzedazao-C

26	501	Hamidazao
	587	Yongchengyuanhong
	764	Linzedazao-T
	765	Dunhuangdazao-T
	766	Anningxiaozao
	813	Fuzao-T
	817	Cuizao
27	82	Dayewuhezao
	555	Neihuangdayewuhe
28	95	Fengmiguan
	598	Dalilinglingzao-T
	615	Dalifengmiguan
	618	Zhongcaobenzao
29	99	Wuhezao-C7
	100	Xiaozao-C2
	216	Xiaozao-C40
	232	C23
	233	Zhanpudazao
30	107	Mayazao
	108	S006
31	110	Bashenghu
	617	Dalibashenghu
32	121	Linglingzao
	183	Jishanbanzao-C
	523	Jishanzaobanzao
33	124	Songxiandazao-C
	601	Binxianjinzao
	662	Songxiandazao-T
34	127	Zhentouzao
	226	Henanlongzao-C
	231	Damayazao-C
	503	Hebeilongzao
	619	Henanlongzao-T
	625	Tengzhoutangzao
	643	Damayazao-T
35	139	Meimizao
	388	BJ30
36	148	Chengwudongzao-T
	644	Chengwudongzao -C
37	149	Liuyuexian-C
	179	C16
	637	Liuyuexian-T
38	154	Tailihong
	499	Zhenpingtailihong
39	171	Xiaozao-C21
	206	Xiaozao-C30
	287	Wanrongfuzao
	387	BJ29

Overall analysis (Cangzhou and Taigu)	40	172	Mamazao-C1
		671	Ludamazao
		675	Malingcui
		691	Mamazao-T
		702	Gagazao-T
	41	173	Gedazao-C1
		638	Gedazao-T
	42	174	Suyuanling-C
		345	Zizao-C2
		614	SS1
		624	Lelingmopanzao
		631	Yuanling-NO.1
		632	Yuanling-NO.2
		635	Suyuanling-T
		694	Yuanling
	43	191	Xiaozao-C27
		248	Luzao-NO.4
	44	196	Kongfucui
		246	Gedazao-C2
		673	Kongfusucuizao
	45	198	Jinai-NO.2
		359	Xuputiansuanzao
	46	201	Lengbaiyu
		741	Leng-4
	47	217	Sanbianchou-C
		549	Puyangsanbianhong
		669	Sanbianchou-T
	48	218	C19
		546	Huizaozhiban-NO.1
		548	Xinzhenghuizao
		559	Xinzhengchangjixinzao
	49	229	Dabailing
		252	C27
	50	234	Daguazao-C
		676	Daguazao-T
		680	Fengjiejidanzao
	51	240	Luzao-NO.11-C1
		340	Luzao-NO.11-C2
	52	244	Luzao-NO.9-C1
		342	Luzao-NO.9-C2
	53	255	Jinsi-NO.4-C
		693	Jinsi-NO.1
	54	274	Beijinghuluzao-C
		411	BJ47
	55	275	Jiaxianchangzao
		292	Nanjingdamuzao-C
	56	278	Heigeda
		606	Binxianheigeda

	894	Binxianhonggeda
57	280	Tengzhouchanghong-C
	626	Tengzhouchanghong-T
58	284	Tianjinkuaizao
	346	Zhishezao
59	288	Yiwuezizao
	888	Shiloushuaizao
60	301	C36
	451	Mifengzao-C
	730	Mifengzao-T
61	304	Hubeiyuanzao
	748	Lingdangzao
62	308	Baodeyouzao-C
	881	Baodeyouzao-T
63	311	C39
	334	C42
	337	Luzao-NO.5
	428	Mizao-C
	449	Xinxing
64	315	Hongzhaoshiyuehong-C
	875	Hongzhaoshiyuehong-T
65	331	Kulianzao-C
	612	Dalizhizao
	613	Xiyingbenzao
66	332	Xiaoguosuanpanzao-C
	725	Xiaosuanpanzao-T
67	336	Dingxiangxingxingzao-C
	657	Pozaozhibian-NO.1
	788	Shanzao
	863	Dingxiangxingxingzao-T
68	343	Lengzao-C
	757	Lengzao-T
69	348	Muzao-C
	550	Yucidamozao
	583	Yanchuantiaozao
	595	Lajiaozao-NO.1
	596	Lajiaozao-NO.2
	666	Wuxingzao
	667	Muzao-T
	677	Shidizao
	815	Hejintiaozao
	872	Pingyaodazao
	876	Xiangfenyazao
	910	Wutaimuzao
70	350	Yanliangxiangzao
	607	Lintongguluzao
71	353	Manaizao
	687	Banzao-T

71	688	Leng-2
	941	Hongcunbaizao
72	374	BJ15
	376	BJ17
	401	BJ56
73	389	BJ32
	415	BJ55
74	397	BJ48
	413	BJ51
	454	Jingzao-40
75	404	BJ34
	405	BJ35
	407	BJ39
	409	BJ43
	414	BJ54
76	412	BJ50
	436	Shanxiwumingzao
77	457	Jidanzao-C3
	575	Shanxihuluzao
78	465	Henan-9
	466	C48
79	472	Lincui-NO.1
	474	Jiaochengtiansuanzao-C
80	477	Hongzhaohuluzao-C
	873	Hongzhaohuluzao-T
81	480	Yuanquzao-C
	840	Yuanquzao-T
82	492	Cangxiantunzizao
	495	Yutianxiaozao
	504	Xianxian-NO.21
	505	Xianxiandaxiaozao
	506	Xianxianmuzao
	510	Zaoqianggutouxiaozao
	511	Xianxianxiaoxiaozao
	521	Cangxianchangxiaozao
	529	Xianxianyuanxiaozao
	538	Gusuxiaozao
	567	Beijingbenzao
	569	Xinzhengdazao
	579	Lejin-NO.3
	580	Lelingwuhe-NO.2
83	493	Cangxianxiaozao
	578	Lejin-NO.4
	581	Lejin-NO.1
84	494	Shulutangzao
	556	Neihuangbianhesuan
	672	Chahuzao-T
	498	Shenxianchuanganhong

85	536	Shenxianchuanganzao
	685	Chuanganzao
86	517	Qingyuandadanzao
	551	Beijingzhuizibai
87	519	Liyiwumingzao
	726	Changzao
	834	Yongjihamazao
88	522	Jinnan-NO.1
	542	Xincaidayuanfeng
89	531	Xianxiansuanzao
	713	Yuanzao
90	547	Xinzhengjixinzao
	571	Xinzhengjiantouhuizao
91	561	Yucinaitouzao
	565	Yucimianzao
92	563	Yucichangmuzao
	699	08-1
	805	Bangchuizao
	831	Linhuang-NO.1
	842	Taigulajiao-NO.1
	844	Taigulajiao-NO.2
	854	Taigulangzao
	869	Pingshunbenzao
	882	Baodexiaozao
	897	Jiaxianchangmuzao
93	584	Guantan-NO.1
	586	Guantan-NO.2
	684	Guantanwang
	849	Xiangfenguantanzao
94	589	Dalimayazao
	599	Binxiansuangedazao
95	593	Puchengdundunzao
	609	Daliyuanzao
	611	Daliganweiba
96	648	Midiancuimuzao
	654	Shaanxiyazao
97	690	Laopozao-T
	905	Baodingyueguang
	917	BeijinggagazaoT2
	923	Beijingmayazao-T
	932	Baozhuangjianzao
98	695	Langjiayuanzao-T
	747	Jixinzao-T
99	705	Qiyuexian
	803	Yongjijidanzao
100	710	Changdazao
	723	Mizao-T
	750	Dahongzao

	101	752	Zhongningxiaozao
		763	Linzexiaozao
	102	770	Wukuzhakexiaozao
		816	Jinudazao
		819	Qingxumoguzao
		833	Jinchang-NO.1-T
		846	Taiguhuping-NO.1
		895	Fupingjidanzao
		898	Jinsi-NO.3-1
		944	Chaoyangwuhezao
		951	Chaoyangjinsimi
	103	772	Kuerlexiaozao
		938	Chaoyangqicuizao
	104	778	Yixianmuzao
		814	Linyibenzao-T
	105	787	Dingxiangxiaozao
		792	Mianmeizao
		801	Tanzao-T
		853	Lichengxiaozao-T
		928	Baozhuangxiaozao
		933	Beijingtaizimu
		937	Suziyumizao
		952	Chaoyangmeixinzao
		961	Jinsi-NO.3-2
	106	790	Wutaimianzao-T1
		859	Pingyaobuluosu
	107	795	Malingsuan
		796	Taiyuanshiyuehong
	108	804	Habazao-T
		810	Jidanzao-T
		812	Bobozao
	109	806	Shouxingzao
		883	Pinglutuntunzao
	110	808	Daling
		809	Xiaolingzao
		839	Pinglujianzao
	111	820	Jiaochengyazao
		862	Taigulinglingzao
	112	832	Taigulonghuzao
		848	Taiguhuping-NO.2
	113	843	Linfenmizao-T
		916	Beijing-31
	114	845	Linfentuanzao
		867	Zhongyangtuanzao-T
	115	850	Hongzhaocuizao
		871	Hongzaoxiaozao-T
	116	891	Huxiantaipingjianzao
		892	Huxiantaipingshuizao

		896	Lintongmalianzao
	117	900	Zhumadianyangjiaozao
		901	Tongbaidazao
	118	909	Wutaimianzao-T2
		912	Wutaicuzao
	119	911	Jingzao-39
		920	Yuetanzao-T2
		962	Xiangjingzao-39
	120	921	Beijingpaopaozao
		935	Changpingdahongpao
	121	922	Changxindianbaizao-2
		924	Changxindianbaizao-1
	122	926	Baozhuangxiaoyuanzao
		936	Chaoyangwanzao
	123	946	Chaoyangxiaopingding
		949	Chaoyangjinlingchangzao
		955	Chaoyanglingzao
		957	Ershijiadazao
		958	Gendedazao

Table S3 Summary of private alleles between Cangzhou and Taigu germplasm repository based on accessions used in this study, excluding the triploid species

Locus	Allele	Frequency	Germplasm preserved units
586	269	0.006	C
586	273	0.002	C
586	295	0.001	C
377	288	0.001	C
377	294	0.006	C
539	227	0.001	C
539	241	0.001	C
539	245	0.004	C
539	249	0.001	C
249	270	0.001	C
249	284	0.002	C
249	288	0.002	C
308	152	0.001	C
308	154	0.008	C
308	186	0.004	C
473	299	0.003	C
584	314	0.001	C
1157	236	0.002	C
501	154	0.004	C
614	271	0.001	C
614	277	0.004	C
614	279	0.003	C
479	230	0.001	C
479	242	0.001	C
479	248	0.002	C
479	250	0.001	C
574	106	0.011	C
574	132	0.001	C
521	238	0.001	C
521	242	0.001	C
521	270	0.001	C
521	272	0.001	C
521	274	0.001	C
286	276	0.001	C
1409	166	0.001	C
1409	168	0.001	C
564	129	0.007	C
564	131	0.001	C
564	137	0.006	C
478	212	0.002	T
1205	173	0.001	T
377	310	0.002	T

377	312	0.003	T
539	255	0.001	T
308	178	0.001	T
308	198	0.003	T
473	289	0.001	T
1157	242	0.002	T
1157	258	0.001	T
614	261	0.001	T
1178	305	0.002	T
479	260	0.002	T
574	120	0.001	T
574	128	0.001	T
521	268	0.002	T
286	298	0.001	T
1409	170	0.001	T
1409	192	0.001	T

Table S4 List of accessions with one or more private alleles based on accessions used in this study, excluding the triploid accessions

Code	Accession name	Allele number	Private allele	Germplasm preserved units
14	Huairoudacuizao	1	BFU0249 (270)	C
18	Shaanxigedazao	1	BFU1157(236)	C
19	Jidanzao-C1	2	BFU0564(129,137)	C
30	Longxuzao	1	BFU1157(236)	C
36	Chaoyangyuanzao	1	BFU0564(131)	C
40	Xuezao	1	BFU0564(129)	C
43	Xiaoyuanzao	1	BFU0614(271)	C
72	Lingbaodazao-C	1	BFU0501(154)	C
80	C10	2	BFU0539(227),BFU1409(166)	C
91	Mayazao-C2	2	BFU0586(273),BFU0286(276)	C
106	S161	2	BFU0574(106),BFU0564(137)	C
111	Cuizaohong	5	BFU0586(269),BFU0377(294),BFU 0521(242),BFU0564(129,137)	C
122	Xiaozao-C8	2	BFU0564(129,137)	C
123	Xiaozao-C9	1	BFU0377(294)	C
126	Yueyazao	1	BFU0377(294)	C
128	Jinsixin-NO.1	1	BFU0614(279)	C
132	Wenxianshatang	2	BFU0473(299),BFU0479(242)	C
144	Xiaozao-C11	1	BFU0479(248)	C
146	Henan-12	3	BFU0586(269),BFU0574(106),BFU 0564(129)	C
157	Linfenmizao-C	1	BFU0521(238)	C
167	Xiaozao-C17	2	BFU0564(129,137)	C
180	Cuzao	3	BFU0501(154),BFU0614(277),BFU 0564(129)	C
185	Xiaozao-C22	1	BFU0584(314)	C
194	C17	2	BFU0586(269),BFU0574(106)	C
242	Luzao-NO.10-C1	1	BFU0308(154)	C
251	Luzao-NO.6	1	BFU0574(106)	C
253	Suizhoudazao	2	BFU0539(241,249)	C
262	BJ11	1	BFU0574(106)	C
264	BJ21	1	BFU0308(186)	C
265	C29	2	BFU0586(269),BFU0377(294)	C
274	Beijinghuluzao-C	2	BFU0539(245),BFU0308(186)	C
293	Jinzao-NO.3	1	BFU0574(132)	C
294	Yuanlizao-C2	1	BFU0308(186)	C
306	C38	1	BFU0249(284)	C
317	Shaanxixiaodundunza	1	BFU0249(288)	C
325	C40	1	BFU0377(294)	C
326	Zhenhuluzao	1	BFU1409(168)	C
330	Lianxianmuzao-C2	1	BFU0586(295)	C
339	Luzao-NO.10-C2	1	BFU0308(152)	C
372	BJ27	1	BFU0521(270)	C

385	BJ10	1	BFU0614(279)	C
389	BJ32	1	BFU0539(245)	C
399	BJ52	1	BFU0614(279)	C
406	BJ38	2	BFU0308(154),BFU0564(137)	C
418	Yuexiangcuizao	1	BFU0614(277)	C
420	Zidantou	1	BFU0308(154)	C
424	Yejiatianzao	1	BFU0479(230)	C
432	Aowang	2	BFU0377(288),BFU0308(154)	C
433	Fupingdazao	1	BFU0308(154)	C
434	Xiaopingding	1	BFU0308(154)	C
459	Jikang-NO.1	1	BFU0473(299)	C
460	Changhong	2	521(272,274)	C
478	Suanzao	1	BFU0479(250)	C
482	Lincui-NO.5	1	BFU0586(273)	C
483	Shaanximianzao-C	1	BFU0586(269)	C
484	Zhishegedazao-C	1	BFU0586(269)	C
486	Xinjiangwumingzao	1	BFU0377(294)	C
488	Shaanxiqiyuexian	1	BFU1178(305)	T
508	Xuechengdongzao	1	BFU0308(178)	T
681	Guchengdongzao	1	BFU0521(268)	T
735	Nanjingdamuzao-T	1	BFU1409(192)	T
738	Mianxuzao	1	BFU0308(198)	T
769	Kashixiaozao	3	BFU0478(212),BFU0377(310),BFU 0574(128)	T
771	Xinjiangxiaoyuanzao	1	BFU0377(310)	T
783	Guangdongbaizao	1	BFU0286(298)	T
786	Guangdongtangzao	1	BFU0479(260)	T
791	Yucijiuyueqing	1	BFU01178(305)	T
800	Lvfendan	1	BFU0377(312)	T
829	Linyizhenzhulongzao	1	BFU01157(258)	T
850	Hongzhaocuizao	1	BFU0308(198)	T
866	Taiguhuluzao-T	1	BFU0377(312)	T
877	Jiaochengduanzao	1	BFU1409(170)	T
879	Jiaochengtiansuanzao-	1	BFU0478(212)	T
904	Hanguohongyan	2	BFU0539(255),BFU0614(261)	T
940	Chaoyangmazao	1	BFU0377(312)	T
947	Chaoyangdajianding	1	BFU0473(289)	T
948	Chaoyangxiaojianding	1	BFU1157(242)	T
953	Chaoyangxiaoyuanling	1	BFU1157(242)	T
959	Chaoyangmopanzao	2	BFU1205(173),BFU0574(120)	T
960	Jinximuzao	1	BFU0521(268)	T

Table S5 List of conserved accessions and their fruit size, the tolerance to fruit cracking		
Accession name	Cracking level	Fruit size
Jishanbanzao	High tolerant	Intermediate
Zhongyangmuzao	High tolerant	Intermediate
Longxuzao	High tolerant	Small
Xuezao	High tolerant	Extremely large
Huanghuadongzao	High tolerant	Intermediate
Wanshuyuanling	High tolerant	Large
Muzao	Tolerant	Intermediate
Jidanzao	Tolerant	Extremely large
Ningyangliuyuexian	Tolerant	Intermediate
Jinzao	Tolerant	Large
Lajiaozao	Tolerance	Intermediate
Wuhezao	Susceptible	Small
Meimizao	Susceptible	Intermediate
Jinsixiaozao	Susceptible	Small
Junzao	Highly Susceptible	Large
Fengmiguan	Highly Susceptible	Small
Mayazao	Highly Susceptible	Intermediate
Xinzhenghuizao	Highly Susceptible	Small
Linfenmizao	Highly Susceptible	Intermediate

Table S6 Information of accessions used in this study, including the code, the accession name, the species and the germplasm preserved units			
**Code**	**Accession name**	**Species**	**Preserved location**
1	Dalilongzao-C	*Ziziphus jujuba Mill. var. jujuba*	C
2	Gutouxiaozao	*Ziziphus jujuba Mill. var. jujuba*	C
3	Yuanlingxiaozao	*Ziziphus jujuba Mill. var. jujuba*	C
4	Wuhezao-C1	*Ziziphus jujuba Mill. var. jujuba*	C
5	Lizao	*Ziziphus jujuba Mill. var. jujuba*	C
6	Dongzao-103	*Ziziphus jujuba Mill. var. jujuba*	C
7	Zaocuiwang	*Ziziphus jujuba Mill. var. jujuba*	C
8	Pozao-C1	*Ziziphus jujuba Mill. var. jujuba*	C
9	Wuhezao-C2	*Ziziphus jujuba Mill. var. jujuba*	C
10	Zanhuangdazao-C	*Ziziphus jujuba Mill. var. jujuba*	C
11	Mayizao-C	*Ziziphus jujuba Mill. var. jujuba*	C
12	Yiwuzao	*Ziziphus jujuba Mill. var. jujuba*	C
13	C50	*Ziziphus jujuba Mill. var. jujuba*	C
14	Huairoudacuizao	*Ziziphus jujuba Mill. var. jujuba*	C
15	S182	*Ziziphus jujuba Mill. var. jujuba*	C
16	Langjiayuanzao-C	*Ziziphus jujuba Mill. var. jujuba*	C
17	Wuhezao-C3	*Ziziphus jujuba Mill. var. jujuba*	C
18	Shaanxigedazao	*Ziziphus jujuba Mill. var. jujuba*	C
19	Jidanzao-C1	*Ziziphus jujuba Mill. var. jujuba*	C
20	Sanlengzao	*Ziziphus jujuba Mill. var. jujuba*	C
21	Manmanzao	*Ziziphus jujuba Mill. var. jujuba*	C
22	C51	*Ziziphus jujuba Mill. var. jujuba*	C
23	C02	*Ziziphus jujuba Mill. var. jujuba*	C
24	C03	*Ziziphus jujuba Mill. var. jujuba*	C
25	Hupingzao-C1	*Ziziphus jujuba Mill. var. jujuba*	C
26	C52	*Ziziphus jujuba Mill. var. jujuba*	C
27	Hupingzao-C2	*Ziziphus jujuba Mill. var. jujuba*	C
28	Popozao	*Ziziphus jujuba Mill. var. jujuba*	C
29	Pingguozao	*Ziziphus jujuba Mill. var. jujuba*	C
30	Longxuzao	*Ziziphus jujuba Mill. var. jujuba*	C
31	Dongzao-70	*Ziziphus jujuba Mill. var. jujuba*	C
32	Wuhezao-C4	*Ziziphus jujuba Mill. var. jujuba*	C
33	C01	*Ziziphus jujuba Mill. var. jujuba*	C
34	Jinsixiaozao-C1	*Ziziphus jujuba Mill. var. jujuba*	C
35	C04	*Ziziphus jujuba Mill. var. jujuba*	C
36	Chaoyangyuanzao	*Ziziphus jujuba Mill. var. jujuba*	C
37	Chahuzao-C	*Ziziphus jujuba Mill. var. jujuba*	C
38	Lianxianmuzao-C1	*Ziziphus jujuba Mill. var. jujuba*	C
39	Shengxianbaipuzao-C	*Ziziphus jujuba Mill. var. jujuba*	C
40	Xuezao	*Ziziphus jujuba Mill. var. jujuba*	C
41	Lengzao-224	*Ziziphus jujuba Mill. var. jujuba*	C
42	Mingshandazao	*Ziziphus jujuba Mill. var. jujuba*	C
43	Xiaoyuanzao	*Ziziphus jujuba Mill. var. jujuba*	C
44	C05	*Ziziphus jujuba Mill. var. jujuba*	C
45	Goutouzao	*Ziziphus jujuba Mill. var. jujuba*	C
46	Dalingzao	*Ziziphus jujuba Mill. var. jujuba*	C
47	Damuzao	*Ziziphus jujuba Mill. var. jujuba*	C
48	Jinsixiaozao-C2	*Ziziphus jujuba Mill. var. jujuba*	C
49	Wutouzao	*Ziziphus jujuba Mill. var. jujuba*	C
50	C06	*Ziziphus jujuba Mill. var. jujuba*	C

51	Henan-4	*Ziziphus jujuba Mill. var. jujuba*	C
52	Jianzao-C1	*Ziziphus jujuba Mill. var. jujuba*	C
53	Banzao-C	*Ziziphus jujuba Mill. var. jujuba*	C
54	Jinzao-C1	*Ziziphus jujuba Mill. var. jujuba*	C
55	Xiaozizao	*Ziziphus jujuba Mill. var. jujuba*	C
56	Wuhezao-C5	*Ziziphus jujuba Mill. var. jujuba*	C
57	Xiaozao-C1	*Ziziphus jujuba Mill. var. jujuba*	C
58	Duanguochanghong	*Ziziphus jujuba Mill. var. jujuba*	C
59	Huluchanghong-C	*Ziziphus jujuba Mill. var. jujuba*	C
60	Xuanchengyuanzao	*Ziziphus jujuba Mill. var. jujuba*	C
61	C07	*Ziziphus jujuba Mill. var. jujuba*	C
62	Shanghaibaipuzao-C	*Ziziphus jujuba Mill. var. jujuba*	C
63	Ruanhezao-C1	*Ziziphus jujuba Mill. var. jujuba*	C
64	Jiebuqi	*Ziziphus jujuba Mill. var. jujuba*	C
65	Rodouzizao	*Ziziphus jujuba Mill. var. jujuba*	C
66	C08	*Ziziphus jujuba Mill. var. jujuba*	C
67	C09	*Ziziphus jujuba Mill. var. jujuba*	C
68	Yuanzao-206	*Ziziphus jujuba Mill. var. jujuba*	C
69	Dasuanzao	*Ziziphus jujuba Mill. var. jujuba*	C
70	Cuizao-37	*Ziziphus jujuba Mill. var. jujuba*	C
71	Dongzao-40	*Ziziphus jujuba Mill. var. jujuba*	C
72	Lingbaodazao-C	*Ziziphus jujuba Mill. var. jujuba*	C
73	Xingguang	*Ziziphus jujuba Mill. var. jujuba*	C
74	Xiangzao-45	*Ziziphus jujuba Mill. var. jujuba*	C
75	Malianxiaozao	*Ziziphus jujuba Mill. var. jujuba*	C
76	Chuanlingzao-C1	*Ziziphus jujuba Mill. var. jujuba*	C
77	Wuhezao-C6	*Ziziphus jujuba Mill. var. jujuba*	C
78	Henan-6	*Ziziphus jujuba Mill. var. jujuba*	C
79	Henan-7	*Ziziphus jujuba Mill. var. jujuba*	C
80	C10	*Ziziphus jujuba Mill. var. jujuba*	C
81	Dunhuangdazao-C	*Ziziphus jujuba Mill. var. jujuba*	C
82	Dayewuhezao	*Ziziphus jujuba Mill. var. jujuba*	C
83	C11	*Ziziphus jujuba Mill. var. jujuba*	C
84	Wanmianzao	*Ziziphus jujuba Mill. var. jujuba*	C
85	C12	*Ziziphus jujuba Mill. var. jujuba*	C
86	Mianxiaozao	*Ziziphus jujuba Mill. var. jujuba*	C
87	Ao-3	*Ziziphus jujuba Mill. var. jujuba*	C
88	Shaanxijidanzao	*Ziziphus jujuba Mill. var. jujuba*	C
89	Jinai-NO.4	*Ziziphus jujuba Mill. var. jujuba*	C
90	Lichengxiaozao-C	*Ziziphus jujuba Mill. var. jujuba*	C
91	Mayazao-C2	*Ziziphus jujuba Mill. var. jujuba*	C
92	Dongzao-38	*Ziziphus jujuba Mill. var. jujuba*	C
93	Xiangfenmuzao-C	*Ziziphus jujuba Mill. var. jujuba*	C
94	Tuanzao	*Ziziphus jujuba Mill. var. jujuba*	C
95	Fengmiguan	*Ziziphus jujuba Mill. var. jujuba*	C
96	Hamazao	*Ziziphus jujuba Mill. var. jujuba*	C
97	Lajiaozao-C1	*Ziziphus jujuba Mill. var. jujuba*	C
98	Zizao-C1	*Ziziphus jujuba Mill. var. jujuba*	C
99	Wuhezao-C7	*Ziziphus jujuba Mill. var. jujuba*	C
100	Xiaozao-C2	*Ziziphus jujuba Mill. var. jujuba*	C
101	Zaoshutangzao	*Ziziphus jujuba Mill. var. jujuba*	C
102	Xiaolizao	*Ziziphus jujuba Mill. var. jujuba*	C
103	Xupujidanzao-C	*Ziziphus jujuba Mill. var. jujuba*	C
104	Wanshuyuanling	*Ziziphus jujuba Mill. var. jujuba*	C

105	Xinmopan	*Ziziphus jujuba Mill. var. jujuba*	C
106	S161	*Ziziphus jujuba Mill. var. jujuba*	C
107	Mayazao	*Ziziphus jujuba Mill. var. jujuba*	C
108	S006	*Ziziphus jujuba Mill. var. jujuba*	C
109	Niujiaozao	*Ziziphus jujuba Mill. var. jujuba*	C
110	Bashenghu	*Ziziphus jujuba Mill. var. jujuba*	C
111	Cuizaohong	*Ziziphus jujuba Mill. var. jujuba*	C
112	Yuanlizao-C1	*Ziziphus jujuba Mill. var. jujuba*	C
113	C13	*Ziziphus jujuba Mill. var. jujuba*	C
114	Xiaozao-C3	*Ziziphus jujuba Mill. var. jujuba*	C
115	Jinmangguo	*Ziziphus jujuba Mill. var. jujuba*	C
116	Suanjixinzao	*Ziziphus jujuba Mill. var. jujuba*	C
117	Xiaozao-C4	*Ziziphus jujuba Mill. var. jujuba*	C
118	Xiaozao-C5	*Ziziphus jujuba Mill. var. jujuba*	C
119	Xiaozao-C6	*Ziziphus jujuba Mill. var. jujuba*	C
120	Xiaozao-C7	*Ziziphus jujuba Mill. var. jujuba*	C
121	Linglingzao	*Ziziphus jujuba Mill. var. jujuba*	C
122	Xiaozao-C8	*Ziziphus jujuba Mill. var. jujuba*	C
123	Xiaozao-C9	*Ziziphus jujuba Mill. var. jujuba*	C
124	Songxiandazao-C	*Ziziphus jujuba Mill. var. jujuba*	C
125	Guangyangdazao	*Ziziphus jujuba Mill. var. jujuba*	C
126	Yueyazao	*Ziziphus jujuba Mill. var. jujuba*	C
127	Zhentouzao	*Ziziphus jujuba Mill. var. jujuba*	C
128	Jinsixin-NO.1	*Ziziphus jujuba Mill. var. jujuba*	C
129	Mopanzao-C	*Ziziphus jujuba Mill. var. jujuba*	C
130	Gagazao-C	*Ziziphus jujuba Mill. var. jujuba*	C
131	Guantanzao	*Ziziphus jujuba Mill. var. jujuba*	C
132	Wenxianshatang	*Ziziphus jujuba Mill. var. jujuba*	C
133	C14	*Ziziphus jujuba Mill. var. jujuba*	C
134	Mantouzao	*Ziziphus jujuba Mill. var. jujuba*	C
135	Hetaowen-C	*Ziziphus jujuba Mill. var. jujuba*	C
136	Wuhezao-C8	*Ziziphus jujuba Mill. var. jujuba*	C
137	Xiaozao-C10	*Ziziphus jujuba Mill. var. jujuba*	C
138	Buluosu	*Ziziphus jujuba Mill. var. jujuba*	C
139	Meimizao	*Ziziphus jujuba Mill. var. jujuba*	C
140	C15	*Ziziphus jujuba Mill. var. jujuba*	C
141	Jidanzao-C2	*Ziziphus jujuba Mill. var. jujuba*	C
142	Tangzao-C1	*Ziziphus jujuba Mill. var. jujuba*	C
143	Shazao	*Ziziphus jujuba Mill. var. jujuba*	C
144	Xiaozao-C11	*Ziziphus jujuba Mill. var. jujuba*	C
145	Xiaozao-C12	*Ziziphus jujuba Mill. var. jujuba*	C
146	Henan-12	*Ziziphus jujuba Mill. var. jujuba*	C
147	Yingbuluo	*Ziziphus jujuba Mill. var. jujuba*	C
148	Chengwudongzao-T	*Ziziphus jujuba Mill. var. jujuba*	C
149	Liuyuexian-C	*Ziziphus jujuba Mill. var. jujuba*	C
150	Maoertou	*Ziziphus jujuba Mill. var. jujuba*	C
151	Pozao-C2	*Ziziphus jujuba Mill. var. jujuba*	C
152	Mianzao	*Ziziphus jujuba Mill. var. jujuba*	C
153	Chuanlingzao-C2	*Ziziphus jujuba Mill. var. jujuba*	C
154	Tailihong	*Ziziphus jujuba Mill. var. jujuba*	C
155	Xiaomuzao	*Ziziphus jujuba Mill. var. jujuba*	C
156	Qingxuyuanzao-C	*Ziziphus jujuba Mill. var. jujuba*	C
157	Linfenmizao-C	*Ziziphus jujuba Mill. var. jujuba*	C
158	Heiyezao	*Ziziphus jujuba Mill. var. jujuba*	C

159	Manchengtuntunzao	*Ziziphus jujuba Mill. var. jujuba*	C
160	Duanzizao-C	*Ziziphus jujuba Mill. var. jujuba*	C
161	Tangzao-C	*Ziziphus jujuba Mill. var. jujuba*	C
162	Daxuezao	*Ziziphus jujuba Mill. var. jujuba*	C
163	Xiaozao-C13	*Ziziphus jujuba Mill. var. jujuba*	C
164	Xiaozao-C14	*Ziziphus jujuba Mill. var. jujuba*	C
165	Xiaozao-C15	*Ziziphus jujuba Mill. var. jujuba*	C
166	Xiaozao-C16	*Ziziphus jujuba Mill. var. jujuba*	C
167	Xiaozao-C17	*Ziziphus jujuba Mill. var. jujuba*	C
168	Xiaozao-C18	*Ziziphus jujuba Mill. var. jujuba*	C
169	Xiaozao-C19	*Ziziphus jujuba Mill. var. jujuba*	C
170	Xiaozao-C20	*Ziziphus jujuba Mill. var. jujuba*	C
171	Xiaozao-C21	*Ziziphus jujuba Mill. var. jujuba*	C
172	Mamazao-C1	*Ziziphus jujuba Mill. var. jujuba*	C
173	Gedazao-C1	*Ziziphus jujuba Mill. var. jujuba*	C
174	Suyuanling-C	*Ziziphus jujuba Mill. var. jujuba*	C
175	Zhongyangmuzao-C	*Ziziphus jujuba Mill. var. jujuba*	C
176	Linzedazao-C	*Ziziphus jujuba Mill. var. jujuba*	C
177	Gansuxiaokou	*Ziziphus jujuba Mill. var. jujuba*	C
178	Bianshizao	*Ziziphus jujuba Mill. var. jujuba*	C
179	C16	*Ziziphus jujuba Mill. var. jujuba*	C
180	Cuzao	*Ziziphus jujuba Mill. var. jujuba*	C
181	S181	*Ziziphus jujuba Mill. var. jujuba*	C
182	S192	*Ziziphus jujuba Mill. var. jujuba*	C
183	Jishanbanzao-C	*Ziziphus jujuba Mill. var. jujuba*	C
184	Zanyou-NO.1	*Ziziphus jujuba Mill. var. jujuba*	C
185	Xiaozao-C22	*Ziziphus jujuba Mill. var. jujuba*	C
186	Xiaozao-C23	*Ziziphus jujuba Mill. var. jujuba*	C
187	Xiaozao-C24	*Ziziphus jujuba Mill. var. jujuba*	C
188	Xiaozao-C25	*Ziziphus jujuba Mill. var. jujuba*	C
189	Xiaozao-C26	*Ziziphus jujuba Mill. var. jujuba*	C
190	Dongzao-100	*Ziziphus jujuba Mill. var. jujuba*	C
191	Xiaozao-C27	*Ziziphus jujuba Mill. var. jujuba*	C
192	Guazao-C1	*Ziziphus jujuba Mill. var. jujuba*	C
193	Zanhuangzao	*Ziziphus jujuba Mill. var. jujuba*	C
194	C17	*Ziziphus jujuba Mill. var. jujuba*	C
195	Changmuzao-C	*Ziziphus jujuba Mill. var. jujuba*	C
196	Kongfucui	*Ziziphus jujuba Mill. var. jujuba*	C
197	Jinsixin-NO.2	*Ziziphus jujuba Mill. var. jujuba*	C
198	Jinai-NO.2	*Ziziphus jujuba Mill. var. jujuba*	C
199	C18	*Ziziphus jujuba Mill. var. jujuba*	C
200	Jinai-NO.3	*Ziziphus jujuba Mill. var. jujuba*	C
201	Lengbaiyu	*Ziziphus jujuba Mill. var. jujuba*	C
202	Zanyou-NO.2	*Ziziphus jujuba Mill. var. jujuba*	C
203	Zanyou-NO.3	*Ziziphus jujuba Mill. var. jujuba*	C
204	Xiaozao-C28	*Ziziphus jujuba Mill. var. jujuba*	C
205	Xiaozao-C29	*Ziziphus jujuba Mill. var. jujuba*	C
206	Xiaozao-C30	*Ziziphus jujuba Mill. var. jujuba*	C
207	Xiaozao-C31	*Ziziphus jujuba Mill. var. jujuba*	C
208	Xiaozao-C32	*Ziziphus jujuba Mill. var. jujuba*	C
209	Xiaozao-C33	*Ziziphus jujuba Mill. var. jujuba*	C
210	Xiaozao-C34	*Ziziphus jujuba Mill. var. jujuba*	C
211	Xiaozao-C35	*Ziziphus jujuba Mill. var. jujuba*	C
212	Xiaozao-C36	*Ziziphus jujuba Mill. var. jujuba*	C

213	Xiaozao-C37	*Ziziphus jujuba Mill. var. jujuba*	C
214	Xiaozao-C38	*Ziziphus jujuba Mill. var. jujuba*	C
215	Xiaozao-C39	*Ziziphus jujuba Mill. var. jujuba*	C
216	Xiaozao-C40	*Ziziphus jujuba Mill. var. jujuba*	C
217	Sanbianchou-C	*Ziziphus jujuba Mill. var. jujuba*	C
218	C19	*Ziziphus jujuba Mill. var. jujuba*	C
219	C20	*Ziziphus jujuba Mill. var. jujuba*	C
220	Huizao	*Ziziphus jujuba Mill. var. jujuba*	C
221	C21	*Ziziphus jujuba Mill. var. jujuba*	C
222	Yazao-C176	*Ziziphus jujuba Mill. var. jujuba*	C
223	Budaizao	*Ziziphus jujuba Mill. var. jujuba*	C
224	Shanxitedage	*Ziziphus jujuba Mill. var. jujuba*	C
225	Beijingmayazao-C	*Ziziphus jujuba Mill. var. jujuba*	C
226	Henanlongzao-C	*Ziziphus jujuba Mill. var. jujuba*	C
227	Jinai-NO.1	*Ziziphus jujuba Mill. var. jujuba*	C
228	Wuhe-NO.1	*Ziziphus jujuba Mill. var. jujuba*	C
229	Dabailing	*Ziziphus jujuba Mill. var. jujuba*	C
230	C22	*Ziziphus jujuba Mill. var. jujuba*	C
231	Damayazao-C	*Ziziphus jujuba Mill. var. jujuba*	C
232	C23	*Ziziphus jujuba Mill. var. jujuba*	C
233	Zhanpudazao	*Ziziphus jujuba Mill. var. jujuba*	C
234	Daguazao-C	*Ziziphus jujuba Mill. var. jujuba*	C
235	Luzao-NO.8-C1	*Ziziphus jujuba Mill. var. jujuba*	C
236	C24	*Ziziphus jujuba Mill. var. jujuba*	C
237	C25	*Ziziphus jujuba Mill. var. jujuba*	C
238	Hengshuibianzhi	*Ziziphus jujuba Mill. var. jujuba*	C
239	Luzao-NO.3	*Ziziphus jujuba Mill. var. jujuba*	C
240	Luzao-NO.11-C1	*Ziziphus jujuba Mill. var. jujuba*	C
241	C26	*Ziziphus jujuba Mill. var. jujuba*	C
242	Luzao-NO.10-C1	*Ziziphus jujuba Mill. var. jujuba*	C
243	Luzao-NO.1	*Ziziphus jujuba Mill. var. jujuba*	C
244	Luzao-NO.9-C1	*Ziziphus jujuba Mill. var. jujuba*	C
245	Shishengmopan	*Ziziphus jujuba Mill. var. jujuba*	C
246	Gedazao-C2	*Ziziphus jujuba Mill. var. jujuba*	C
247	Yueyacui	*Ziziphus jujuba Mill. var. jujuba*	C
248	Luzao-NO.4	*Ziziphus jujuba Mill. var. jujuba*	C
249	Luzao-NO.7	*Ziziphus jujuba Mill. var. jujuba*	C
250	Laopozao-C	*Ziziphus jujuba Mill. var. jujuba*	C
251	Luzao-NO.6	*Ziziphus jujuba Mill. var. jujuba*	C
252	C27	*Ziziphus jujuba Mill. var. jujuba*	C
253	Suizhoudazao	*Ziziphus jujuba Mill. var. jujuba*	C
254	Zhongqiusucui	*Ziziphus jujuba Mill. var. jujuba*	C
255	Jinsi-NO.4-C	*Ziziphus jujuba Mill. var. jujuba*	C
256	Beijingyingbuluo	*Ziziphus jujuba Mill. var. jujuba*	C
257	Guazao-C2	*Ziziphus jujuba Mill. var. jujuba*	C
258	Wuhezao-C9	*Ziziphus jujuba Mill. var. jujuba*	C
259	C28	*Ziziphus jujuba Mill. var. jujuba*	C
260	Muzaokanglie-NO.1	*Ziziphus jujuba Mill. var. jujuba*	C
261	Wumingzao	*Ziziphus jujuba Mill. var. jujuba*	C
262	BJ11	*Ziziphus jujuba Mill. var. jujuba*	C
263	BJ18	*Ziziphus jujuba Mill. var. jujuba*	C
264	BJ21	*Ziziphus jujuba Mill. var. jujuba*	C
265	C29	*Ziziphus jujuba Mill. var. jujuba*	C
266	Jinlingyuanzao	*Ziziphus jujuba Mill. var. jujuba*	C

267	Fucuimi	*Ziziphus jujuba Mill. var. jujuba*	C
268	Baohulu	*Ziziphus jujuba Mill. var. jujuba*	C
269	C30	*Ziziphus jujuba Mill. var. jujuba*	C
270	Sanbianhong	*Ziziphus jujuba Mill. var. jujuba*	C
271	C31	*Ziziphus jujuba Mill. var. jujuba*	C
272	Qitoubai	*Ziziphus jujuba Mill. var. jujuba*	C
273	Laohuyan	*a Mill. var. spinosa (Bunge) Hu ex H. F*	C
274	Beijinghuluzao-C	*Ziziphus jujuba Mill. var. jujuba*	C
275	Jiaxianchangzao	*Ziziphus jujuba Mill. var. jujuba*	C
276	C32	*Ziziphus jujuba Mill. var. jujuba*	C
277	Shandonglongzao	*Ziziphus jujuba Mill. var. jujuba*	C
278	Heigeda	*Ziziphus jujuba Mill. var. jujuba*	C
279	C33	*Ziziphus jujuba Mill. var. jujuba*	C
280	Tengzhouchanghong-C	*Ziziphus jujuba Mill. var. jujuba*	C
281	Dalishuizao	*Ziziphus jujuba Mill. var. jujuba*	C
282	Jinchang-NO.2	*Ziziphus jujuba Mill. var. jujuba*	C
283	Beibeixiaozao-C	*Ziziphus jujuba Mill. var. jujuba*	C
284	Tianjinkuaizao	*Ziziphus jujuba Mill. var. jujuba*	C
285	Qianlingdazao-C	*Ziziphus jujuba Mill. var. jujuba*	C
286	Yingluozao-C	*Ziziphus jujuba Mill. var. jujuba*	C
287	Wanrongfuzao	*Ziziphus jujuba Mill. var. jujuba*	C
288	Yiwuezizao	*Ziziphus jujuba Mill. var. jujuba*	C
289	Pinglubangchuizao	*Ziziphus jujuba Mill. var. jujuba*	C
290	Xinzhengzaohong	*Ziziphus jujuba Mill. var. jujuba*	C
291	Jikang	*Ziziphus jujuba Mill. var. jujuba*	C
292	Nanjingdamuzao-C	*Ziziphus jujuba Mill. var. jujuba*	C
293	Jinzao-NO.3	*Ziziphus jujuba Mill. var. jujuba*	C
294	Yuanlizao-C2	*Ziziphus jujuba Mill. var. jujuba*	C
295	Linyuzao	*Ziziphus jujuba Mill. var. jujuba*	C
296	Xiaosicui	*Ziziphus jujuba Mill. var. jujuba*	C
297	C34	*Ziziphus jujuba Mill. var. jujuba*	C
298	Tengzhoudamaya	*Ziziphus jujuba Mill. var. jujuba*	C
299	Hongzhaoxiaozao-C	*Ziziphus jujuba Mill. var. jujuba*	C
300	C35	*Ziziphus jujuba Mill. var. jujuba*	C
301	C36	*Ziziphus jujuba Mill. var. jujuba*	C
302	C37	*Ziziphus jujuba Mill. var. jujuba*	C
303	Guangyang-NO.2	*Ziziphus jujuba Mill. var. jujuba*	C
304	Hubeiyuanzao	*Ziziphus jujuba Mill. var. jujuba*	C
305	Xishuangxiaozao-C	*Ziziphus jujuba Mill. var. jujuba*	C
306	C38	*Ziziphus jujuba Mill. var. jujuba*	C
307	Shaoguanbaizao	*Ziziphus jujuba Mill. var. jujuba*	C
308	Baodeyouzao-C	*Ziziphus jujuba Mill. var. jujuba*	C
309	Youhezao-C	*Ziziphus jujuba Mill. var. jujuba*	C
310	Fengyuancui	*Ziziphus jujuba Mill. var. jujuba*	C
311	C39	*Ziziphus jujuba Mill. var. jujuba*	C
312	Yunnan-NO.2-C	*Ziziphus jujuba Mill. var. jujuba*	C
313	Lincui-NO.2	*Ziziphus jujuba Mill. var. jujuba*	C
314	Xuyiyanlaihong	*Ziziphus jujuba Mill. var. jujuba*	C
315	Hongzhaoshiyuehong-C	*Ziziphus jujuba Mill. var. jujuba*	C
316	Xuanchengjianzao-C	*Ziziphus jujuba Mill. var. jujuba*	C
317	Shaanxixiaodundunzao	*Ziziphus jujuba Mill. var. jujuba*	C
318	Fucuihong	*Ziziphus jujuba Mill. var. jujuba*	C
319	Xiyaoyaozaozao	*Ziziphus jujuba Mill. var. jujuba*	C
320	Zhongmoubenzao	*Ziziphus jujuba Mill. var. jujuba*	C

321	Habazao-C	*Ziziphus jujuba Mill. var. jujuba*	C
322	Hejinjinzao-C	*Ziziphus jujuba Mill. var. jujuba*	C
323	Akesuxiaozao-C	*Ziziphus jujuba Mill. var. jujuba*	C
324	Dalilinglingzao-C	*Ziziphus jujuba Mill. var. jujuba*	C
325	C40	*Ziziphus jujuba Mill. var. jujuba*	C
326	Zhenhuluzao	*Ziziphus jujuba Mill. var. jujuba*	C
327	Taiguduanzizao-C	*Ziziphus jujuba Mill. var. jujuba*	C
328	Shanxigedazao	*Ziziphus jujuba Mill. var. jujuba*	C
329	Zhongyangtuanzao-C	*Ziziphus jujuba Mill. var. jujuba*	C
330	Lianxianmuzao-C2	*Ziziphus jujuba Mill. var. jujuba*	C
331	Kulianzao-C	*Ziziphus jujuba Mill. var. jujuba*	C
332	Xiaoguosuanpanzao-C	*Ziziphus jujuba Mill. var. jujuba*	C
333	C41	*Ziziphus jujuba Mill. var. jujuba*	C
334	C42	*Ziziphus jujuba Mill. var. jujuba*	C
335	C43	*Ziziphus jujuba Mill. var. jujuba*	C
336	Dingxiangxingxingzao	*Ziziphus jujuba Mill. var. jujuba*	C
337	Luzao-NO.5	*Ziziphus jujuba Mill. var. jujuba*	C
338	Luzao-NO.2	*Ziziphus jujuba Mill. var. jujuba*	C
339	Luzao-NO.10-C2	*Ziziphus jujuba Mill. var. jujuba*	C
340	Luzao-NO.11-C2	*Ziziphus jujuba Mill. var. jujuba*	C
341	Luzao-NO.8-C2	*Ziziphus jujuba Mill. var. jujuba*	C
342	Luzao-NO.9-C2	*Ziziphus jujuba Mill. var. jujuba*	C
343	Lengzao-C	*Ziziphus jujuba Mill. var. jujuba*	C
344	Jinxuan-NO.1	*Ziziphus jujuba Mill. var. jujuba*	C
345	Zizao-C2	*Ziziphus jujuba Mill. var. jujuba*	C
346	Zhishezao	*Ziziphus jujuba Mill. var. jujuba*	C
347	Yanliangcuizao	*Ziziphus jujuba Mill. var. jujuba*	C
348	Muzao-C	*Ziziphus jujuba Mill. var. jujuba*	C
349	Shanxilizao	*Ziziphus jujuba Mill. var. jujuba*	C
350	Yanliangxiangzao	*Ziziphus jujuba Mill. var. jujuba*	C
351	Jingzao-60	*Ziziphus jujuba Mill. var. jujuba*	C
352	Miguanxin-NO.1	*Ziziphus jujuba Mill. var. jujuba*	C
353	Manaizao	*Ziziphus jujuba Mill. var. jujuba*	C
354	Fuzao-C	*Ziziphus jujuba Mill. var. jujuba*	C
355	Xiaoyazao	*Ziziphus jujuba Mill. var. jujuba*	C
356	Yingshanhong	*Ziziphus jujuba Mill. var. jujuba*	C
357	Dongzao	*Ziziphus jujuba Mill. var. jujuba*	C
358	Wanronggedazao	*Ziziphus jujuba Mill. var. jujuba*	C
359	Xuputiansuanzao	*Ziziphus jujuba Mill. var. jujuba*	C
360	Wuxiangtianzao	*Ziziphus jujuba Mill. var. jujuba*	C
361	C44	*Ziziphus jujuba Mill. var. jujuba*	C
362	Hunanchangzao	*Ziziphus jujuba Mill. var. jujuba*	C
363	Linyibenzao-C	*Ziziphus jujuba Mill. var. jujuba*	C
364	BJ22	*Ziziphus jujuba Mill. var. jujuba*	C
365	BJ12	*Ziziphus jujuba Mill. var. jujuba*	C
366	BJ23	*Ziziphus jujuba Mill. var. jujuba*	C
367	BJ26	*Ziziphus jujuba Mill. var. jujuba*	C
368	BJ24	*Ziziphus jujuba Mill. var. jujuba*	C
369	BJ19	*Ziziphus jujuba Mill. var. jujuba*	C
370	BJ20	*Ziziphus jujuba Mill. var. jujuba*	C
371	BJ25	*Ziziphus jujuba Mill. var. jujuba*	C
372	BJ27	*Ziziphus jujuba Mill. var. jujuba*	C
373	BJ01	*Ziziphus jujuba Mill. var. jujuba*	C
374	BJ15	*Ziziphus jujuba Mill. var. jujuba*	C

375	BJ16	*Ziziphus jujuba Mill. var. jujuba*	C
376	BJ17	*Ziziphus jujuba Mill. var. jujuba*	C
377	BJ08	*Ziziphus jujuba Mill. var. jujuba*	C
378	BJ05	*Ziziphus jujuba Mill. var. jujuba*	C
379	BJ06	*Ziziphus jujuba Mill. var. jujuba*	C
380	BJ02	*Ziziphus jujuba Mill. var. jujuba*	C
381	BJ03	*Ziziphus jujuba Mill. var. jujuba*	C
382	BJ04	*Ziziphus jujuba Mill. var. jujuba*	C
383	BJ09	*Ziziphus jujuba Mill. var. jujuba*	C
384	BJ07	*Ziziphus jujuba Mill. var. jujuba*	C
385	BJ10	*Ziziphus jujuba Mill. var. jujuba*	C
386	BJ13	*Ziziphus jujuba Mill. var. jujuba*	C
387	BJ29	*Ziziphus jujuba Mill. var. jujuba*	C
388	BJ30	*Ziziphus jujuba Mill. var. jujuba*	C
389	BJ32	*Ziziphus jujuba Mill. var. jujuba*	C
390	BJ33	*Ziziphus jujuba Mill. var. jujuba*	C
391	BJ36	*Ziziphus jujuba Mill. var. jujuba*	C
392	BJ37	*Ziziphus jujuba Mill. var. jujuba*	C
393	BJ40	*Ziziphus jujuba Mill. var. jujuba*	C
394	BJ41	*Ziziphus jujuba Mill. var. jujuba*	C
395	BJ44	*Ziziphus jujuba Mill. var. jujuba*	C
396	BJ45	*Ziziphus jujuba Mill. var. jujuba*	C
397	BJ48	*Ziziphus jujuba Mill. var. jujuba*	C
398	BJ49	*Ziziphus jujuba Mill. var. jujuba*	C
399	BJ52	*Ziziphus jujuba Mill. var. jujuba*	C
400	BJ53	*Ziziphus jujuba Mill. var. jujuba*	C
401	BJ56	*Ziziphus jujuba Mill. var. jujuba*	C
402	BJ28	*Ziziphus jujuba Mill. var. jujuba*	C
403	BJ31	*Ziziphus jujuba Mill. var. jujuba*	C
404	BJ34	*Ziziphus jujuba Mill. var. jujuba*	C
405	BJ35	*Ziziphus jujuba Mill. var. jujuba*	C
406	BJ38	*Ziziphus jujuba Mill. var. jujuba*	C
407	BJ39	*Ziziphus jujuba Mill. var. jujuba*	C
408	BJ42	*Ziziphus jujuba Mill. var. jujuba*	C
409	BJ43	*Ziziphus jujuba Mill. var. jujuba*	C
410	BJ46	*Ziziphus jujuba Mill. var. jujuba*	C
411	BJ47	*Ziziphus jujuba Mill. var. jujuba*	C
412	BJ50	*Ziziphus jujuba Mill. var. jujuba*	C
413	BJ51	*Ziziphus jujuba Mill. var. jujuba*	C
414	BJ54	*Ziziphus jujuba Mill. var. jujuba*	C
415	BJ55	*Ziziphus jujuba Mill. var. jujuba*	C
416	C45	*Ziziphus jujuba Mill. var. jujuba*	C
417	Daguoshengzhouchan gzao	*Ziziphus jujuba Mill. var. jujuba*	C
418	Yuexiangcuizao	*Ziziphus jujuba Mill. var. jujuba*	C
419	Yuanguoshengzhoubai[illegible]	*Ziziphus jujuba Mill. var. jujuba*	C
420	Zidantou	*Ziziphus jujuba Mill. var. jujuba*	C
421	Xiaoguoshengzhouyua nzao	*Ziziphus jujuba Mill. var. jujuba*	C
422	Qiguzao	*Ziziphus jujuba Mill. var. jujuba*	C
423	Niunaizao	*Ziziphus jujuba Mill. var. jujuba*	C
424	Yejiatianzao	*Ziziphus jujuba Mill. var. jujuba*	C
425	Jinchang-NO.1-C	*Ziziphus jujuba Mill. var. jujuba*	C
426	Ruanhezao-C2	*Ziziphus jujuba Mill. var. jujuba*	C

427	C46	*Ziziphus jujuba Mill. var. jujuba*	C
428	Mizao-C	*Ziziphus jujuba Mill. var. jujuba*	C
429	Jianzao-C2	*Ziziphus jujuba Mill. var. jujuba*	C
430	BJ57	*Ziziphus jujuba Mill. var. jujuba*	C
431	BJ14	*Ziziphus jujuba Mill. var. jujuba*	C
432	Aowang	*Ziziphus jujuba Mill. var. jujuba*	C
433	Fupingdazao	*Ziziphus jujuba Mill. var. jujuba*	C
434	Xiaopingding	*Ziziphus jujuba Mill. var. jujuba*	C
435	Lucui	*Ziziphus jujuba Mill. var. jujuba*	C
436	Shanxiwumingzao	*Ziziphus jujuba Mill. var. jujuba*	C
437	Xuanlingzao-C	*Ziziphus jujuba Mill. var. jujuba*	C
438	Bopicui	*Ziziphus jujuba Mill. var. jujuba*	C
439	C47	*Ziziphus jujuba Mill. var. jujuba*	C
440	Tanzao-C	*Ziziphus jujuba Mill. var. jujuba*	C
441	Maguzao	*Ziziphus jujuba Mill. var. jujuba*	C
442	ZhongguoShengzhouchangzao	*Ziziphus jujuba Mill. var. jujuba*	C
443	Zanyu	*Ziziphus jujuba Mill. var. jujuba*	C
444	Liuyuehong	*Ziziphus jujuba Mill. var. jujuba*	C
445	Hunanwumingzao	*Ziziphus jujuba Mill. var. jujuba*	C
446	Changzicui	*Ziziphus jujuba Mill. var. jujuba*	C
447	Hunanlongxuzao	*Ziziphus jujuba Mill. var. jujuba*	C
448	Hunantailihong	*Ziziphus jujuba Mill. var. jujuba*	C
449	Xinxing	*Ziziphus jujuba Mill. var. jujuba*	C
450	Zanjing	*Ziziphus jujuba Mill. var. jujuba*	C
451	Mifengzao-C	*Ziziphus jujuba Mill. var. jujuba*	C
452	Lingzao-C	*Ziziphus jujuba Mill. var. jujuba*	C
453	Daguoshengzhouyuanzao	*Ziziphus jujuba Mill. var. jujuba*	C
454	Jingzao-40	*Ziziphus jujuba Mill. var. jujuba*	C
455	Dapingding	*Ziziphus jujuba Mill. var. jujuba*	C
456	Junzao	*Ziziphus jujuba Mill. var. jujuba*	C
457	Jidanzao-C3	*Ziziphus jujuba Mill. var. jujuba*	C
458	Jiangchuang-NO.1	*Ziziphus jujuba Mill. var. jujuba*	C
459	Jikang-NO.1	*Ziziphus jujuba Mill. var. jujuba*	C
460	Changhong	*Ziziphus jujuba Mill. var. jujuba*	C
461	Jixinzao-C	*Ziziphus jujuba Mill. var. jujuba*	C
462	Hupingzao-C3	*Ziziphus jujuba Mill. var. jujuba*	C
463	Lajiaozao-C2	*Ziziphus jujuba Mill. var. jujuba*	C
464	Xiaozao-C41	*Ziziphus jujuba Mill. var. jujuba*	C
465	Henan-9	*Ziziphus jujuba Mill. var. jujuba*	C
466	C48	*Ziziphus jujuba Mill. var. jujuba*	C
467	C49	*Ziziphus jujuba Mill. var. jujuba*	C
468	Mamazao-C2	*Ziziphus jujuba Mill. var. jujuba*	C
469	Wuhezao-C10	*Ziziphus jujuba Mill. var. jujuba*	C
470	Longzao	*Ziziphus jujuba Mill. var. jujuba*	C
471	Jinzao-C2	*Ziziphus jujuba Mill. var. jujuba*	C
472	Lincui-NO.1	*Ziziphus jujuba Mill. var. jujuba*	C
473	Suancuiwang	*Ziziphus jujuba Mill. var. jujuba*	C
474	Jiaochengtiansuanzao-	*Ziziphus jujuba Mill. var. jujuba*	C
475	Kuduanzao	*Ziziphus jujuba Mill. var. jujuba*	C
476	Xupuliuyuezao	*Ziziphus jujuba Mill. var. jujuba*	C
477	Hongzhaohuluzao-C	*Ziziphus jujuba Mill. var. jujuba*	C
478	Suanzao	*ba Mill. var. spinosa (Bunge) Hu ex H. F*	C

479	Dongzaoerdai	*Ziziphus jujuba Mill. var. jujuba*	C
480	Yanchuanbaizao-C	*Ziziphus jujuba Mill. var. jujuba*	C
481	Yuanquzao-C	*Ziziphus jujuba Mill. var. jujuba*	C
482	Lincui-NO.5	*Ziziphus jujuba Mill. var. jujuba*	C
483	Shaanximianzao-C	*Ziziphus jujuba Mill. var. jujuba*	C
484	Zhishegedazao-C	*Ziziphus jujuba Mill. var. jujuba*	C
485	Taiguhuluzao-C	*Ziziphus jujuba Mill. var. jujuba*	C
486	Xinjiangwumingzao	*Ziziphus jujuba Mill. var. jujuba*	C
487	Tianzao	*Ziziphus jujuba Mill. var. jujuba*	C
488	Shaanxiqiyuexian	*Ziziphus jujuba Mill. var. jujuba*	T
489	Taigushenglizao	*Ziziphus jujuba Mill. var. jujuba*	T
490	Lichengchuanlingzao	*Ziziphus jujuba Mill. var. jujuba*	T
491	Xinledazao	*Ziziphus jujuba Mill. var. jujuba*	T
492	Cangxiantunzizao	*Ziziphus jujuba Mill. var. jujuba*	T
493	Cangxianxiaozao	*Ziziphus jujuba Mill. var. jujuba*	T
494	Shulutangzao	*Ziziphus jujuba Mill. var. jujuba*	T
495	Yutianxiaozao	*Ziziphus jujuba Mill. var. jujuba*	T
496	Zanhuangchangzao	*Ziziphus jujuba Mill. var. jujuba*	T
497	Jinxianmuzao	*Ziziphus jujuba Mill. var. jujuba*	T
498	Shenxianchuanganhon	*Ziziphus jujuba Mill. var. jujuba*	T
499	Zhenpingtailihong	*Ziziphus jujuba Mill. var. jujuba*	T
500	Linyilajiao	*Ziziphus jujuba Mill. var. jujuba*	T
501	Hamidazao	*Ziziphus jujuba Mill. var. jujuba*	T
502	Xianxianlajiaozao	*Ziziphus jujuba Mill. var. jujuba*	T
503	Hebeilongzao	*Ziziphus jujuba Mill. var. jujuba*	T
504	Xianxian-NO.21	*Ziziphus jujuba Mill. var. jujuba*	T
505	Xianxiandaxiaozao	*Ziziphus jujuba Mill. var. jujuba*	T
506	Xianxianmuzao	*Ziziphus jujuba Mill. var. jujuba*	T
507	Hebei-NO.13	*Ziziphus jujuba Mill. var. jujuba*	T
508	Xuechengdongzao	*Ziziphus jujuba Mill. var. jujuba*	T
509	Zaoqiangdongzao	*Ziziphus jujuba Mill. var. jujuba*	T
510	Zaoqianggutouxiaozao	*Ziziphus jujuba Mill. var. jujuba*	T
511	Xianxianxiaoxiaozao	*Ziziphus jujuba Mill. var. jujuba*	T
512	Zaoqiangcuizao	*Ziziphus jujuba Mill. var. jujuba*	T
513	Linyiyazao	*Ziziphus jujuba Mill. var. jujuba*	T
514	Yunchengmianzao	*Ziziphus jujuba Mill. var. jujuba*	T
515	Yunchenghamazao	*Ziziphus jujuba Mill. var. jujuba*	T
516	Yunchengcuizao	*Ziziphus jujuba Mill. var. jujuba*	T
517	Qingyuandadanzao	*Ziziphus jujuba Mill. var. jujuba*	T
518	Hetaowen-T	*Ziziphus jujuba Mill. var. jujuba*	T
519	Liyiwumingzao	*Ziziphus jujuba Mill. var. jujuba*	T
520	Zaozhuanggongzao	*Ziziphus jujuba Mill. var. jujuba*	T
521	Cangxianchangxiaozao	*Ziziphus jujuba Mill. var. jujuba*	T
522	Jinnan-NO.1	*Ziziphus jujuba Mill. var. jujuba*	T
523	Jishanbanzao	*Ziziphus jujuba Mill. var. jujuba*	T
524	Zaoqiangshazao	*Ziziphus jujuba Mill. var. jujuba*	T
525	Jinsitedazao	*Ziziphus jujuba Mill. var. jujuba*	T
526	Xianxianmianzao	*Ziziphus jujuba Mill. var. jujuba*	T
527	Hanguoyuechu	*Ziziphus jujuba Mill. var. jujuba*	T
528	Hanguowudeng	*Ziziphus jujuba Mill. var. jujuba*	T
529	Xianxianyuanxiaozao	*Ziziphus jujuba Mill. var. jujuba*	T
530	Cangxianjinsixiaozao	*Ziziphus jujuba Mill. var. jujuba*	T
531	Xianxiansuanzao	*a Mill. var. spinosa (Bunge) Hu ex H. F*	T
532	Taigumeixinhe	*Ziziphus jujuba Mill. var. jujuba*	T

533	Taigumeixinhong	*Ziziphus jujuba Mill. var. jujuba*	T
534	Guoyanhong	*Ziziphus jujuba Mill. var. jujuba*	T
535	Taigudasuanzao	*ba Mill. var. spinosa (Bunge) Hu ex H. F*	T
536	Shenxianchuanganzao	*Ziziphus jujuba Mill. var. jujuba*	T
537	Taiguhupingzao	*Ziziphus jujuba Mill. var. jujuba*	T
538	Gusuxiaozao	*Ziziphus jujuba Mill. var. jujuba*	T
539	Jiaochengjunzao	*Ziziphus jujuba Mill. var. jujuba*	T
540	Xinzhengsuzao	*Ziziphus jujuba Mill. var. jujuba*	T
541	Xinzhengjidanzao	*Ziziphus jujuba Mill. var. jujuba*	T
542	Xincaidayuanfeng	*Ziziphus jujuba Mill. var. jujuba*	T
543	Zanhuangdazao-T	*Ziziphus jujuba Mill. var. jujuba*	T
544	Shandonglizao	*Ziziphus jujuba Mill. var. jujuba*	T
545	Beijingjidanzao	*Ziziphus jujuba Mill. var. jujuba*	T
546	Huizaozhiban-NO.1	*Ziziphus jujuba Mill. var. jujuba*	T
547	Xinzhengjixinzao	*Ziziphus jujuba Mill. var. jujuba*	T
548	Xinzhenghuizao	*Ziziphus jujuba Mill. var. jujuba*	T
549	Puyangsanbianhong	*Ziziphus jujuba Mill. var. jujuba*	T
550	Yucidamozao	*Ziziphus jujuba Mill. var. jujuba*	T
551	Beijingzhuizibai	*Ziziphus jujuba Mill. var. jujuba*	T
552	Lingbaodazao-T	*Ziziphus jujuba Mill. var. jujuba*	T
553	Zhenpingguangyangzao	*Ziziphus jujuba Mill. var. jujuba*	T
554	Anyangtuanzao	*Ziziphus jujuba Mill. var. jujuba*	T
555	Neihuangdayewuhe	*Ziziphus jujuba Mill. var. jujuba*	T
556	Neihuangbianhesuan	*Ziziphus jujuba Mill. var. jujuba*	T
557	Linxianwutouzao	*Ziziphus jujuba Mill. var. jujuba*	T
558	Huanghuadongzao	*Ziziphus jujuba Mill. var. jujuba*	T
559	Xinzhengchangjixinzao	*Ziziphus jujuba Mill. var. jujuba*	T
560	Xinzhengdamaya	*Ziziphus jujuba Mill. var. jujuba*	T
561	Yucinaitouzao	*Ziziphus jujuba Mill. var. jujuba*	T
562	Yuciwanhongzao	*Ziziphus jujuba Mill. var. jujuba*	T
563	Yucichangmuzao	*Ziziphus jujuba Mill. var. jujuba*	T
564	Yunchengpopozao	*Ziziphus jujuba Mill. var. jujuba*	T
565	Yucimianzao	*Ziziphus jujuba Mill. var. jujuba*	T
566	Yuciyazao	*Ziziphus jujuba Mill. var. jujuba*	T
567	Beijingbenzao	*Ziziphus jujuba Mill. var. jujuba*	T
568	Xinzhengqitoubai	*Ziziphus jujuba Mill. var. jujuba*	T
569	Xinzhengdazao	*Ziziphus jujuba Mill. var. jujuba*	T
570	Xinzhengxiaoyuanzao	*Ziziphus jujuba Mill. var. jujuba*	T
571	Xinzhengjiantouhuizao	*Ziziphus jujuba Mill. var. jujuba*	T
572	Puyangtangzao	*Ziziphus jujuba Mill. var. jujuba*	T
573	Linyilizao	*Ziziphus jujuba Mill. var. jujuba*	T
574	Yanchuandieyazao	*Ziziphus jujuba Mill. var. jujuba*	T
575	Shanxihuluzao	*Ziziphus jujuba Mill. var. jujuba*	T
576	Hupingzao-NO.2	*Ziziphus jujuba Mill. var. jujuba*	T
577	Hupingzao-NO.1	*Ziziphus jujuba Mill. var. jujuba*	T
578	Lejin-NO.4	*Ziziphus jujuba Mill. var. jujuba*	T
579	Lejin-NO.3	*Ziziphus jujuba Mill. var. jujuba*	T
580	Lelingwuhe-NO.2	*Ziziphus jujuba Mill. var. jujuba*	T
581	Lejin-NO.1	*Ziziphus jujuba Mill. var. jujuba*	T
582	Lejin-NO.2	*Ziziphus jujuba Mill. var. jujuba*	T
583	Yanchuantiaozao	*Ziziphus jujuba Mill. var. jujuba*	T
584	Guantan-NO.1	*Ziziphus jujuba Mill. var. jujuba*	T
585	Yongchengchanghong	*Ziziphus jujuba Mill. var. jujuba*	T
586	Guantan-NO.2	*Ziziphus jujuba Mill. var. jujuba*	T

587	Yongchengyuanhong	*Ziziphus jujuba Mill. var. jujuba*	T
588	Dalixiaodundunzao	*Ziziphus jujuba Mill. var. jujuba*	T
589	Dalimayazao	*Ziziphus jujuba Mill. var. jujuba*	T
590	Dalijidanzao	*Ziziphus jujuba Mill. var. jujuba*	T
591	Puchengmianzao	*Ziziphus jujuba Mill. var. jujuba*	T
592	Puchengyuanlizao	*Ziziphus jujuba Mill. var. jujuba*	T
593	Puchengdundunzao	*Ziziphus jujuba Mill. var. jujuba*	T
594	Shaanximianzao-T	*Ziziphus jujuba Mill. var. jujuba*	T
595	Lajiaozao-NO.1	*Ziziphus jujuba Mill. var. jujuba*	T
596	Lajiaozao-NO.2	*Ziziphus jujuba Mill. var. jujuba*	T
597	Dalilongzao-T	*Ziziphus jujuba Mill. var. jujuba*	T
598	Dalilinglingzao-T	*Ziziphus jujuba Mill. var. jujuba*	T
599	Binxiansuangedazao	*Ziziphus jujuba Mill. var. jujuba*	T
600	Qianlingdazao-T	*Ziziphus jujuba Mill. var. jujuba*	T
601	Binxianjinzao	*Ziziphus jujuba Mill. var. jujuba*	T
602	Jinzao-NO.1	*Ziziphus jujuba Mill. var. jujuba*	T
603	Dalipachizao	*Ziziphus jujuba Mill. var. jujuba*	T
604	Binxianyuanzao	*Ziziphus jujuba Mill. var. jujuba*	T
605	Zaoqiangmianianxiaozao	*Ziziphus jujuba Mill. var. jujuba*	T
606	Binxianheigeda	*Ziziphus jujuba Mill. var. jujuba*	T
607	Lintongguluzao	*Ziziphus jujuba Mill. var. jujuba*	T
608	Binxianshuizao	*Ziziphus jujuba Mill. var. jujuba*	T
609	Daliyuanzao	*Ziziphus jujuba Mill. var. jujuba*	T
610	Puchengzhishezao	*Ziziphus jujuba Mill. var. jujuba*	T
611	Daliganweiba	*Ziziphus jujuba Mill. var. jujuba*	T
612	Dalizhizao	*Ziziphus jujuba Mill. var. jujuba*	T
613	Xiyingbenzao	*Ziziphus jujuba Mill. var. jujuba*	T
614	SS1	*Ziziphus jujuba Mill. var. jujuba*	T
615	Dalifengmiguan	*Ziziphus jujuba Mill. var. jujuba*	T
616	Daligedazao	*Ziziphus jujuba Mill. var. jujuba*	T
617	Dalibashenghu	*Ziziphus jujuba Mill. var. jujuba*	T
618	Zhongcaobenzao	*Ziziphus jujuba Mill. var. jujuba*	T
619	Henanlongzao-T	*Ziziphus jujuba Mill. var. jujuba*	T
620	Shaanxinaizao	*Ziziphus jujuba Mill. var. jujuba*	T
621	Yanchuanbaizao-T	*Ziziphus jujuba Mill. var. jujuba*	T
622	Dabaizao	*Ziziphus jujuba Mill. var. jujuba*	T
623	Yanchuanniunaicuizao	*Ziziphus jujuba Mill. var. jujuba*	T
624	Lelingmopanzao	*Ziziphus jujuba Mill. var. jujuba*	T
625	Tengzhoutangzao	*Ziziphus jujuba Mill. var. jujuba*	T
626	Tengzhouchanghong-T	*Ziziphus jujuba Mill. var. jujuba*	T
627	Lelingxiaozao	*Ziziphus jujuba Mill. var. jujuba*	T
628	Lelingwuhexiaozao	*Ziziphus jujuba Mill. var. jujuba*	T
629	Hebeiwuhezao	*Ziziphus jujuba Mill. var. jujuba*	T
630	Liaochengyuanlingzao	*Ziziphus jujuba Mill. var. jujuba*	T
631	Yuanling-NO.1	*Ziziphus jujuba Mill. var. jujuba*	T
632	Yuanling-NO.2	*Ziziphus jujuba Mill. var. jujuba*	T
633	Shishengdongzao	*Ziziphus jujuba Mill. var. jujuba*	T
634	Dacuizao	*Ziziphus jujuba Mill. var. jujuba*	T
635	Suyuanling-T	*Ziziphus jujuba Mill. var. jujuba*	T
636	Changmuzao-T	*Ziziphus jujuba Mill. var. jujuba*	T
637	Liuyuexian-T	*Ziziphus jujuba Mill. var. jujuba*	T
638	Gedazao-T	*Ziziphus jujuba Mill. var. jujuba*	T
639	Lingzao-T	*Ziziphus jujuba Mill. var. jujuba*	T
640	Huluchanghong-T	*Ziziphus jujuba Mill. var. jujuba*	T

641	Sanbianse	*Ziziphus jujuba Mill. var. jujuba*	T
642	Jiuyuexian	*Ziziphus jujuba Mill. var. jujuba*	T
643	Damayazao-T	*Ziziphus jujuba Mill. var. jujuba*	T
644	Chengwudongzao -C	*Ziziphus jujuba Mill. var. jujuba*	T
645	Xuanlingzao-T	*Ziziphus jujuba Mill. var. jujuba*	T
646	Shaanxixiaoyuanzao	*Ziziphus jujuba Mill. var. jujuba*	T
647	Huluzao	*Ziziphus jujuba Mill. var. jujuba*	T
648	Midiancuimuzao	*Ziziphus jujuba Mill. var. jujuba*	T
649	Linqinzao	*Ziziphus jujuba Mill. var. jujuba*	T
650	Xiyaozao	*Ziziphus jujuba Mill. var. jujuba*	T
651	Yangnaizao	*Ziziphus jujuba Mill. var. jujuba*	T
652	Jiaxianbaizao	*Ziziphus jujuba Mill. var. jujuba*	T
653	Heyanglinglingzao	*Ziziphus jujuba Mill. var. jujuba*	T
654	Shaanxiyazao	*Ziziphus jujuba Mill. var. jujuba*	T
655	Zheshegedazao-T	*Ziziphus jujuba Mill. var. jujuba*	T
656	Puchengjinzao	*Ziziphus jujuba Mill. var. jujuba*	T
657	Pozaozhibian-NO.1	*Ziziphus jujuba Mill. var. jujuba*	T
658	Jiuyueqing	*Ziziphus jujuba Mill. var. jujuba*	T
659	Henanxiaozao	*Ziziphus jujuba Mill. var. jujuba*	T
660	Lingbaoling-NO.1	*Ziziphus jujuba Mill. var. jujuba*	T
661	Guoxingshibingzhuang	*Ziziphus jujuba Mill. var. jujuba*	T
662	Songxiandazao-T	*Ziziphus jujuba Mill. var. jujuba*	T
663	Henanpingguozao	*Ziziphus jujuba Mill. var. jujuba*	T
664	Malingzao	*Ziziphus jujuba Mill. var. jujuba*	T
665	Lajiaozao-T	*Ziziphus jujuba Mill. var. jujuba*	T
666	Wuxingzao	*Ziziphus jujuba Mill. var. jujuba*	T
667	Muzao-T	*Ziziphus jujuba Mill. var. jujuba*	T
668	Mopanzao-T	*Ziziphus jujuba Mill. var. jujuba*	T
669	Sanbianchou-T	*Ziziphus jujuba Mill. var. jujuba*	T
670	Luodihong	*Ziziphus jujuba Mill. var. jujuba*	T
671	Ludamazao	*Ziziphus jujuba Mill. var. jujuba*	T
672	Chahuzao-T	*Ziziphus jujuba Mill. var. jujuba*	T
673	Kongfusucuizao	*Ziziphus jujuba Mill. var. jujuba*	T
674	Houtouzao	*Ziziphus jujuba Mill. var. jujuba*	T
675	Malingcui	*Ziziphus jujuba Mill. var. jujuba*	T
676	Daguazao-T	*Ziziphus jujuba Mill. var. jujuba*	T
677	Shidizao	*Ziziphus jujuba Mill. var. jujuba*	T
678	Lushibingzao	*Ziziphus jujuba Mill. var. jujuba*	T
679	Beibeixiaozao-T	*Ziziphus jujuba Mill. var. jujuba*	T
680	Fengjiejidanzao	*Ziziphus jujuba Mill. var. jujuba*	T
681	Guchengdongzao	*Ziziphus jujuba Mill. var. jujuba*	T
682	Yucituanzao	*Ziziphus jujuba Mill. var. jujuba*	T
683	Zanhuangtedage	*Ziziphus jujuba Mill. var. jujuba*	T
684	Guantanwang	*Ziziphus jujuba Mill. var. jujuba*	T
685	Chuanganzao	*Ziziphus jujuba Mill. var. jujuba*	T
686	Zilingdan	*Ziziphus jujuba Mill. var. jujuba*	T
687	Banzao-T	*Ziziphus jujuba Mill. var. jujuba*	T
688	Leng-2	*Ziziphus jujuba Mill. var. jujuba*	T
689	Leng-3	*Ziziphus jujuba Mill. var. jujuba*	T
690	Laopozao-T	*Ziziphus jujuba Mill. var. jujuba*	T
691	Mamazao-T	*Ziziphus jujuba Mill. var. jujuba*	T
692	Qingyunxiaolizao	*Ziziphus jujuba Mill. var. jujuba*	T
693	Jinsi-NO.1	*Ziziphus jujuba Mill. var. jujuba*	T
694	Yuanling	*Ziziphus jujuba Mill. var. jujuba*	T

695	Langjiayuanzao-T	*Ziziphus jujuba Mill. var. jujuba*	T
696	Miyunxiaozao	*Ziziphus jujuba Mill. var. jujuba*	T
697	Yingluozao-T	*Ziziphus jujuba Mill. var. jujuba*	T
698	Jingxixiaobaizao	*Ziziphus jujuba Mill. var. jujuba*	T
699	08-1	*Ziziphus jujuba Mill. var. jujuba*	T
700	Lizao-NO.1	*Ziziphus jujuba Mill. var. jujuba*	T
701	Tianjin-NO.1	*Ziziphus jujuba Mill. var. jujuba*	T
702	Gagazao-T	*Ziziphus jujuba Mill. var. jujuba*	T
703	Minzao	*Ziziphus jujuba Mill. var. jujuba*	T
704	Erqiuzao	*Ziziphus jujuba Mill. var. jujuba*	T
705	Qiyuexian	*Ziziphus jujuba Mill. var. jujuba*	T
706	Kuaizao	*Ziziphus jujuba Mill. var. jujuba*	T
707	Kangtouzao	*Ziziphus jujuba Mill. var. jujuba*	T
708	Zhenzhuzao	*Ziziphus jujuba Mill. var. jujuba*	T
709	Xiangjianzao	*Ziziphus jujuba Mill. var. jujuba*	T
710	Changdazao	*Ziziphus jujuba Mill. var. jujuba*	T
711	Tangzao-T	*Ziziphus jujuba Mill. var. jujuba*	T
712	Xupujidanzao-T	*Ziziphus jujuba Mill. var. jujuba*	T
713	Yuanzao	*Ziziphus jujuba Mill. var. jujuba*	T
714	Tiansuanzao	*Ziziphus jujuba Mill. var. jujuba*	T
715	Ruchengzao	*Ziziphus jujuba Mill. var. jujuba*	T
716	Daguosuanpanzao	*Ziziphus jujuba Mill. var. jujuba*	T
717	Chengtuozao-T	*Ziziphus jujuba Mill. var. jujuba*	T
718	Guanyinzao	*Ziziphus jujuba Mill. var. jujuba*	T
719	Xiangyuanzao	*Ziziphus jujuba Mill. var. jujuba*	T
720	Xiangmuzao	*Ziziphus jujuba Mill. var. jujuba*	T
721	Xiangzao	*Ziziphus jujuba Mill. var. jujuba*	T
722	Yanzao	*Ziziphus jujuba Mill. var. jujuba*	T
723	Mizao-T	*Ziziphus jujuba Mill. var. jujuba*	T
724	Chengchuizao	*Ziziphus jujuba Mill. var. jujuba*	T
725	Xiaosuanpanzao-T	*Ziziphus jujuba Mill. var. jujuba*	T
726	Changzao	*Ziziphus jujuba Mill. var. jujuba*	T
727	Binlangzao	*Ziziphus jujuba Mill. var. jujuba*	T
728	Liuyuezao	*Ziziphus jujuba Mill. var. jujuba*	T
729	Xupushibingzao	*Ziziphus jujuba Mill. var. jujuba*	T
730	Mifengzao-T	*Ziziphus jujuba Mill. var. jujuba*	T
731	Shatangzao	*Ziziphus jujuba Mill. var. jujuba*	T
732	Bopizao	*Ziziphus jujuba Mill. var. jujuba*	T
733	Lanximazao	*Ziziphus jujuba Mill. var. jujuba*	T
734	Shengxianbaipuzao-T	*Ziziphus jujuba Mill. var. jujuba*	T
735	Nanjingdamuzao-T	*Ziziphus jujuba Mill. var. jujuba*	T
736	Ezizao	*Ziziphus jujuba Mill. var. jujuba*	T
737	Suantianzao	*Ziziphus jujuba Mill. var. jujuba*	T
738	Mianxuzao	*Ziziphus jujuba Mill. var. jujuba*	T
739	Yiwudazao	*Ziziphus jujuba Mill. var. jujuba*	T
740	Mazao	*Ziziphus jujuba Mill. var. jujuba*	T
741	Leng-4	*Ziziphus jujuba Mill. var. jujuba*	T
742	Shaizao	*Ziziphus jujuba Mill. var. jujuba*	T
743	Mayizao-T	*Ziziphus jujuba Mill. var. jujuba*	T
744	Wanniunaizao	*Ziziphus jujuba Mill. var. jujuba*	T
745	Xuanchengjianzao-T	*Ziziphus jujuba Mill. var. jujuba*	T
746	Wanmutouzao	*Ziziphus jujuba Mill. var. jujuba*	T
747	Jixinzao-T	*Ziziphus jujuba Mill. var. jujuba*	T
748	Lingdangzao	*Ziziphus jujuba Mill. var. jujuba*	T

749	Gansudongzao	*Ziziphus jujuba Mill. var. jujuba*	T
750	Dahongzao	*Ziziphus jujuba Mill. var. jujuba*	T
751	Diaolingzao	*Ziziphus jujuba Mill. var. jujuba*	T
752	Zhongningxiaozao	*Ziziphus jujuba Mill. var. jujuba*	T
753	Nanjingyazao	*Ziziphus jujuba Mill. var. jujuba*	T
754	Nanjingzao	*Ziziphus jujuba Mill. var. jujuba*	T
755	Shuituanzao	*Ziziphus jujuba Mill. var. jujuba*	T
756	Wangcun-NO.1	*Ziziphus jujuba Mill. var. jujuba*	T
757	Lengzao-T	*Ziziphus jujuba Mill. var. jujuba*	T
758	Yanlaihong	*Ziziphus jujuba Mill. var. jujuba*	T
759	Shanghaibaipuzao-T	*Ziziphus jujuba Mill. var. jujuba*	T
760	Guanyangduanzao	*Ziziphus jujuba Mill. var. jujuba*	T
761	Guanyangchangzao	*Ziziphus jujuba Mill. var. jujuba*	T
762	Diaodiaopo	*Ziziphus jujuba Mill. var. jujuba*	T
763	Linzexiaozao	*Ziziphus jujuba Mill. var. jujuba*	T
764	Linzedazao-T	*Ziziphus jujuba Mill. var. jujuba*	T
765	Dunhuangdazao-T	*Ziziphus jujuba Mill. var. jujuba*	T
766	Anningxiaozao	*Ziziphus jujuba Mill. var. jujuba*	T
767	Minqinxiaozao	*Ziziphus jujuba Mill. var. jujuba*	T
768	Ningxiachangzao	*Ziziphus jujuba Mill. var. jujuba*	T
769	Kashixiaozao	*Ziziphus jujuba Mill. var. jujuba*	T
770	Wukuzhakexiaozao	*Ziziphus jujuba Mill. var. jujuba*	T
771	Xinjiangxiaoyuanzao	*Ziziphus jujuba Mill. var. jujuba*	T
772	Kuerlexiaozao	*Ziziphus jujuba Mill. var. jujuba*	T
773	Changyuanzao	*Ziziphus jujuba Mill. var. jujuba*	T
774	Zanxindazao	*Ziziphus jujuba Mill. var. jujuba*	T
775	Yuancuizao	*Ziziphus jujuba Mill. var. jujuba*	T
776	Akesuxiaozao-T	*Ziziphus jujuba Mill. var. jujuba*	T
777	Zunyitianzao	*Ziziphus jujuba Mill. var. jujuba*	T
778	Yixianmuzao	*Ziziphus jujuba Mill. var. jujuba*	T
779	Xishuangxiaozao-T	*Ziziphus jujuba Mill. var. jujuba*	T
780	Yunnan-NO.2-T	*Ziziphus jujuba Mill. var. jujuba*	T
781	Kunmingzao	*Ziziphus jujuba Mill. var. jujuba*	T
782	Kulianzao-T	*Ziziphus jujuba Mill. var. jujuba*	T
783	Guangdongbaizao	*Ziziphus jujuba Mill. var. jujuba*	T
784	Guangdongdazao	*Ziziphus jujuba Mill. var. jujuba*	T
785	Guangdongmuzao	*Ziziphus jujuba Mill. var. jujuba*	T
786	Guangdongtangzao	*Ziziphus jujuba Mill. var. jujuba*	T
787	Dingxiangxiaozao	*Ziziphus jujuba Mill. var. jujuba*	T
788	Shanzao	*Ziziphus jujuba Mill. var. jujuba*	T
789	Youhezao-T	*Ziziphus jujuba Mill. var. jujuba*	T
790	Wutaimianzao-T1	*Ziziphus jujuba Mill. var. jujuba*	T
791	Yucijiuyueqing	*Ziziphus jujuba Mill. var. jujuba*	T
792	Mianmeizao	*Ziziphus jujuba Mill. var. jujuba*	T
793	Maohouzao	*Ziziphus jujuba Mill. var. jujuba*	T
794	Qiutuanzao	*Ziziphus jujuba Mill. var. jujuba*	T
795	Malingsuan	*Ziziphus jujuba Mill. var. jujuba*	T
796	Taiyuanshiyuehong	*Ziziphus jujuba Mill. var. jujuba*	T
797	Hejinjinzao-T	*Ziziphus jujuba Mill. var. jujuba*	T
798	Taiyuanyuanzao	*Ziziphus jujuba Mill. var. jujuba*	T
799	Taiyuanchangzao	*Ziziphus jujuba Mill. var. jujuba*	T
800	Lvfendan	*Ziziphus jujuba Mill. var. jujuba*	T
801	Tanzao-T	*Ziziphus jujuba Mill. var. jujuba*	T
802	Yazao	*Ziziphus jujuba Mill. var. jujuba*	T

803	Yongjijidanzao	*Ziziphus jujuba Mill. var. jujuba*	T
804	Habazao-T	*Ziziphus jujuba Mill. var. jujuba*	T
805	Bangchuizao	*Ziziphus jujuba Mill. var. jujuba*	T
806	Shouxingzao	*Ziziphus jujuba Mill. var. jujuba*	T
807	Mugedazao	*Ziziphus jujuba Mill. var. jujuba*	T
808	Daling	*Ziziphus jujuba Mill. var. jujuba*	T
809	Xiaolingzao	*Ziziphus jujuba Mill. var. jujuba*	T
810	Jidanzao-T	*Ziziphus jujuba Mill. var. jujuba*	T
811	Hejinshuizao	*Ziziphus jujuba Mill. var. jujuba*	T
812	Bobozao	*Ziziphus jujuba Mill. var. jujuba*	T
813	Fuzao-T	*Ziziphus jujuba Mill. var. jujuba*	T
814	Linyibenzao-T	*Ziziphus jujuba Mill. var. jujuba*	T
815	Hejintiaozao	*Ziziphus jujuba Mill. var. jujuba*	T
816	Jinudazao	*Ziziphus jujuba Mill. var. jujuba*	T
817	Cuizao	*Ziziphus jujuba Mill. var. jujuba*	T
818	Bolicui	*Ziziphus jujuba Mill. var. jujuba*	T
819	Qingxumoguzao	*Ziziphus jujuba Mill. var. jujuba*	T
820	Jiaochengyazao	*Ziziphus jujuba Mill. var. jujuba*	T
821	Jan-01	*Ziziphus jujuba Mill. var. jujuba*	T
822	Feb-01	*Ziziphus jujuba Mill. var. jujuba*	T
823	Mar-01	*Ziziphus jujuba Mill. var. jujuba*	T
824	May-01	*Ziziphus jujuba Mill. var. jujuba*	T
825	Apr-01	*Ziziphus jujuba Mill. var. jujuba*	T
826	Jan-03	*Ziziphus jujuba Mill. var. jujuba*	T
827	Qingxupingguozao	*Ziziphus jujuba Mill. var. jujuba*	T
828	Damozao	*Ziziphus jujuba Mill. var. jujuba*	T
829	Linyizhenzhulongzao	*Ziziphus jujuba Mill. var. jujuba*	T
830	Jinzandazao	*Ziziphus jujuba Mill. var. jujuba*	T
831	Linhuang-NO.1	*Ziziphus jujuba Mill. var. jujuba*	T
832	Taigulonghuzao	*Ziziphus jujuba Mill. var. jujuba*	T
833	Jinchang-NO.1-T	*Ziziphus jujuba Mill. var. jujuba*	T
834	Yongjihamazao	*Ziziphus jujuba Mill. var. jujuba*	T
835	Yunchengxiangzao	*Ziziphus jujuba Mill. var. jujuba*	T
836	Duanzhizao-T	*Ziziphus jujuba Mill. var. jujuba*	T
837	Cuitian-NO.1	*Ziziphus jujuba Mill. var. jujuba*	T
838	Jishanbanzao-T	*Ziziphus jujuba Mill. var. jujuba*	T
839	Pinglujianzao	*Ziziphus jujuba Mill. var. jujuba*	T
840	Yuanquzao-T	*Ziziphus jujuba Mill. var. jujuba*	T
841	Xiaxianziyuanzao	*Ziziphus jujuba Mill. var. jujuba*	T
842	Taigulajiao-NO.1	*Ziziphus jujuba Mill. var. jujuba*	T
843	Linfenmizao-T	*Ziziphus jujuba Mill. var. jujuba*	T
844	Taigulajiao-NO.2	*Ziziphus jujuba Mill. var. jujuba*	T
845	Linfentuanzao	*Ziziphus jujuba Mill. var. jujuba*	T
846	Taiguhuping-NO.1	*Ziziphus jujuba Mill. var. jujuba*	T
847	Xiangfenyuanzao	*Ziziphus jujuba Mill. var. jujuba*	T
848	Taiguhuping-NO.2	*Ziziphus jujuba Mill. var. jujuba*	T
849	Xiangfenguantanzao	*Ziziphus jujuba Mill. var. jujuba*	T
850	Hongzhaocuizao	*Ziziphus jujuba Mill. var. jujuba*	T
851	Xiaxianyuancuizao	*Ziziphus jujuba Mill. var. jujuba*	T
852	Linfenzhenhulu	*Ziziphus jujuba Mill. var. jujuba*	T
853	Lichengxiaozao-T	*Ziziphus jujuba Mill. var. jujuba*	T
854	Taigulangzao	*Ziziphus jujuba Mill. var. jujuba*	T
855	Pingshunjunzao	*Ziziphus jujuba Mill. var. jujuba*	T
856	Taiguhupingsuan	*Ziziphus jujuba Mill. var. jujuba*	T

857	Zhongyangmuzao-T	*Ziziphus jujuba Mill. var. jujuba*	T
858	Taigumeimizao	*Ziziphus jujuba Mill. var. jujuba*	T
859	Pingyaobuluosu	*Ziziphus jujuba Mill. var. jujuba*	T
860	Taigujixinmizao	*Ziziphus jujuba Mill. var. jujuba*	T
861	Qingxuyuanzao-T	*Ziziphus jujuba Mill. var. jujuba*	T
862	Taigulinglingzao	*Ziziphus jujuba Mill. var. jujuba*	T
863	Dingxiangxingxingzao-T	*Ziziphus jujuba Mill. var. jujuba*	T
864	Taiguheiyezao	*Ziziphus jujuba Mill. var. jujuba*	T
865	Lichengdamazao	*Ziziphus jujuba Mill. var. jujuba*	T
866	Taiguhuluzao-T	*Ziziphus jujuba Mill. var. jujuba*	T
867	Zhongyangtuanzao-T	*Ziziphus jujuba Mill. var. jujuba*	T
868	Taigudundunzao	*Ziziphus jujuba Mill. var. jujuba*	T
869	Pingshunbenzao	*Ziziphus jujuba Mill. var. jujuba*	T
870	Taiguduanzizao-T	*Ziziphus jujuba Mill. var. jujuba*	T
871	Hongzaoxiaozao-T	*Ziziphus jujuba Mill. var. jujuba*	T
872	Pingyaodazao	*Ziziphus jujuba Mill. var. jujuba*	T
873	Hongzhaohuluzao-T	*Ziziphus jujuba Mill. var. jujuba*	T
874	Pingyaokuduanzao	*Ziziphus jujuba Mill. var. jujuba*	T
875	Hongzhaoshiyuehong-T	*Ziziphus jujuba Mill. var. jujuba*	T
876	Xiangfenyazao	*Ziziphus jujuba Mill. var. jujuba*	T
877	Jiaochengduanzao	*Ziziphus jujuba Mill. var. jujuba*	T
878	Xiangfenmuzao-T	*Ziziphus jujuba Mill. var. jujuba*	T
879	Jiaochengtiansuanzao-T	*Ziziphus jujuba Mill. var. jujuba*	T
880	Wenshuishazao	*Ziziphus jujuba Mill. var. jujuba*	T
881	Baodeyouzao-T	*Ziziphus jujuba Mill. var. jujuba*	T
882	Baodexiaozao	*Ziziphus jujuba Mill. var. jujuba*	T
883	Pinglutuntunzao	*Ziziphus jujuba Mill. var. jujuba*	T
884	Liuguanzao	*Ziziphus jujuba Mill. var. jujuba*	T
885	Jishanchangzao	*Ziziphus jujuba Mill. var. jujuba*	T
886	Jishanyuanzao	*Ziziphus jujuba Mill. var. jujuba*	T
887	Shiloucuizao	*Ziziphus jujuba Mill. var. jujuba*	T
888	Shiloushuaizao	*Ziziphus jujuba Mill. var. jujuba*	T
889	Lingzao-T1	*Ziziphus jujuba Mill. var. jujuba*	T
890	Zuiguifei	*Ziziphus jujuba Mill. var. jujuba*	T
891	Huxiantaipingjianzao	*Ziziphus jujuba Mill. var. jujuba*	T
892	Huxiantaipingshuizao	*Ziziphus jujuba Mill. var. jujuba*	T
893	Binxiansuzao	*Ziziphus jujuba Mill. var. jujuba*	T
894	Binxianhonggeda	*Ziziphus jujuba Mill. var. jujuba*	T
895	Fupingjidanzao	*Ziziphus jujuba Mill. var. jujuba*	T
896	Lintongmalianzao	*Ziziphus jujuba Mill. var. jujuba*	T
897	Jiaxianchangmuzao	*Ziziphus jujuba Mill. var. jujuba*	T
898	Jinsi-NO.3-1	*Ziziphus jujuba Mill. var. jujuba*	T
899	Jinsi-NO.4-T	*Ziziphus jujuba Mill. var. jujuba*	T
900	Zhumadianyangjiaozao	*Ziziphus jujuba Mill. var. jujuba*	T
901	Tongbaidazao	*Ziziphus jujuba Mill. var. jujuba*	T
902	Hanguofuzao	*Ziziphus jujuba Mill. var. jujuba*	T
903	Hanguojinxiu	*Ziziphus jujuba Mill. var. jujuba*	T
904	Hanguohongyan	*Ziziphus jujuba Mill. var. jujuba*	T
905	Baodingyueguang	*Ziziphus jujuba Mill. var. jujuba*	T
906	Jiangchuang-NO.2	*Ziziphus jujuba Mill. var. jujuba*	T
907	Wutaiwudizao	*Ziziphus jujuba Mill. var. jujuba*	T
908	Zhuluyouyouzao	*Ziziphus jujuba Mill. var. jujuba*	T
909	Wutaimianzao-T2	*Ziziphus jujuba Mill. var. jujuba*	T
910	Wutaimuzao	*Ziziphus jujuba Mill. var. jujuba*	T

911	Jingzao-39-T1	*Ziziphus jujuba Mill. var. jujuba*	T
912	Wutaicuzao	*Ziziphus jujuba Mill. var. jujuba*	T
913	Beijinghuluzao-T	*Ziziphus jujuba Mill. var. jujuba*	T
914	Beijingdacuizao	*Ziziphus jujuba Mill. var. jujuba*	T
915	Beijinggagazao-T1	*Ziziphus jujuba Mill. var. jujuba*	T
916	Beijing-31	*Ziziphus jujuba Mill. var. jujuba*	T
917	BeijinggagazaoT2	*Ziziphus jujuba Mill. var. jujuba*	T
918	Yuetanzao-T1	*Ziziphus jujuba Mill. var. jujuba*	T
919	Beijinghuashengzao	*Ziziphus jujuba Mill. var. jujuba*	T
920	Yuetanzao-T2	*Ziziphus jujuba Mill. var. jujuba*	T
921	Beijingpaopaozao	*Ziziphus jujuba Mill. var. jujuba*	T
922	Changxindianbaizao-2	*Ziziphus jujuba Mill. var. jujuba*	T
923	Beijingmayazao-T	*Ziziphus jujuba Mill. var. jujuba*	T
924	Changxindianbaizao-1	*Ziziphus jujuba Mill. var. jujuba*	T
925	Beijingheiyaozizao	*Ziziphus jujuba Mill. var. jujuba*	T
926	Baozhuangxiaoyuanza[illegible]	*Ziziphus jujuba Mill. var. jujuba*	T
927	Beijingxiaomizao	*Ziziphus jujuba Mill. var. jujuba*	T
928	Baozhuangxiaozao	*Ziziphus jujuba Mill. var. jujuba*	T
929	Beijingsuanzao	*Ziziphus jujuba Mill. var. jujuba*	T
930	Baozhuangdalingzao	*Ziziphus jujuba Mill. var. jujuba*	T
931	Beijingsuzao	*Ziziphus jujuba Mill. var. jujuba*	T
932	Baozhuangjianzao	*Ziziphus jujuba Mill. var. jujuba*	T
933	Beijingtaizimu	*Ziziphus jujuba Mill. var. jujuba*	T
934	Beijinglaohuyan	[illegible]*a Mill. var. spinosa (Bunge) Hu ex H. F*[illegible]	T
935	Changpingdahongpao	*Ziziphus jujuba Mill. var. jujuba*	T
936	Chaoyangwanzao	*Ziziphus jujuba Mill. var. jujuba*	T
937	Suziyumizao	*Ziziphus jujuba Mill. var. jujuba*	T
938	Chaoyangqicuizao	*Ziziphus jujuba Mill. var. jujuba*	T
939	Huairoucuizao	*Ziziphus jujuba Mill. var. jujuba*	T
940	Chaoyangmazao	*Ziziphus jujuba Mill. var. jujuba*	T
941	Hongcunbaizao	*Ziziphus jujuba Mill. var. jujuba*	T
942	Chaoyangmiantaozao	*Ziziphus jujuba Mill. var. jujuba*	T
943	Hongcunbaizao-2	*Ziziphus jujuba Mill. var. jujuba*	T
944	Chaoyangwuhezao	*Ziziphus jujuba Mill. var. jujuba*	T
945	Chaoyangdapingding	*Ziziphus jujuba Mill. var. jujuba*	T
946	Chaoyangxiaopingdin[illegible]	*Ziziphus jujuba Mill. var. jujuba*	T
947	Chaoyangdajianding	*Ziziphus jujuba Mill. var. jujuba*	T
948	Chaoyangxiaojianding	*Ziziphus jujuba Mill. var. jujuba*	T
949	Chaoyangjinlingchang zao	*Ziziphus jujuba Mill. var. jujuba*	T
950	Chaoyangjinlingyuanz[illegible]	*Ziziphus jujuba Mill. var. jujuba*	T
951	Chaoyangjinsimi	*Ziziphus jujuba Mill. var. jujuba*	T
952	Chaoyangmeixinzao	*Ziziphus jujuba Mill. var. jujuba*	T
953	Chaoyangxiaoyuanlin	*Ziziphus jujuba Mill. var. jujuba*	T
954	Chaoyangpingzizao	*Ziziphus jujuba Mill. var. jujuba*	T
955	Chaoyanglingzao	*Ziziphus jujuba Mill. var. jujuba*	T
956	Chaoyangchengtuozao	*Ziziphus jujuba Mill. var. jujuba*	T
957	Ershijiadazao	*Ziziphus jujuba Mill. var. jujuba*	T
958	Gendedazao	*Ziziphus jujuba Mill. var. jujuba*	T
959	Chaoyangmopanzao	*Ziziphus jujuba Mill. var. jujuba*	T
960	Jinximuzao	*Ziziphus jujuba Mill. var. jujuba*	T
961	Jinsi-NO.3-2	*Ziziphus jujuba Mill. var. jujuba*	T
962	Xiangjingzao-39-T2	*Ziziphus jujuba Mill. var. jujuba*	T

Table S7 List of the accessions of sour jujube and triploidy jujube used in this study, including the code, the accession name			
**Category**	**Code**	**Accession name**	**Preserved Location**
**Sour jujube**	273	Laohuyan	C
	478	Suanzao	C
	531	Xianxiansuanzao	T
	535	Taigudasuanzao	T
	934	Beijinglaohuyan	T
**Triploid jujube**	10	Zanhuangdazao-C	C
	29	Pingguozao	C
	184	Zanyou-NO.1	C
	193	Zanhuangzao	C
	202	Zanyou-NO.2	C
	203	Zanyou-NO.3	C
	443	Zanyu	C
	450	Zanjing	C
	496	Zanhuangchangzao	T
	543	Zanhuangdazao-T	T
	663	Henanpingguozao	T
	683	Zanhuangtedazao	T
	774	Zanxindazao	T
	827	Qingxupingguozao	T
	830	Jinzandazao	T
